# Selection of Suitable Reference Genes for qPCR Normalization under Abiotic Stresses and Hormone Stimuli in Carrot Leaves

**DOI:** 10.1371/journal.pone.0117569

**Published:** 2015-02-06

**Authors:** Chang Tian, Qian Jiang, Feng Wang, Guang-Long Wang, Zhi-Sheng Xu, Ai-Sheng Xiong

**Affiliations:** State Key Laboratory of Crop Genetics and Germplasm Enhancement, College of Horticulture, Nanjing Agricultural University, Nanjing, 210095, China; National Institute of Plant Genome Research, INDIA

## Abstract

Carrot, a biennial herb of the Apiaceae family, is among the most important vegetable crops in the world. In this study, nine candidate reference genes (*GAPDH*, *ACTIN, eIF-4α, PP2A, SAND, TIP41, UBQ, EF-1α,* and *TUB*) were cloned from carrot. Carrot plants were subjected to abiotic stresses (heat, cold, salt, and drought) and hormone stimuli (gibberellin, salicylic acid, methyl jasmonate, and abscisic acid). The expression profiles of the candidate reference genes were evaluated in three technical and biological replicates. Real-time qPCR data analyses were performed using three commonly used Excel-based applets namely, BestKeeper, geNorm, and NormFinder. *ACTIN* and *TUB* were the most stable genes identified among all sample groups, but individual analysis revealed changes in their expression profiles. *GAPDH* displayed the maximum stability for most of single stresses. To further validate the suitability of the reference genes identified in this study, the expression profile of *DcDREB-A1* gene (homolog of *AtDREB-A1* gene of *Arabidophsis*) was studied in carrot. The appropriate reference genes were selected that showed stable expression under the different experimental conditions.

## Introduction

Quantitative real-time reverse transcription polymerase chain reaction (qPCR) allows accurate high-throughput RNA quantification over a wide dynamic range at a relatively low cost; this technique has high sensitivity and has been widely used for gene expression analysis [1–4]. Appropriate reference genes could eliminate the discrepancy that may exist in different samples and ensure the accuracy and reliability of the experimental results. Discrepancies may be due to variations in RNA expression levels and the quality and efficiency of reverse transcription. The use of reference genes to measure the temporal and spatial expressions of the target gene is widely acknowledged as a standardized method. In higher plants, suitable internal controls for gene expression studies have been recognized for pepper [5], rice [6], *Arabidopsis thaliana* [7], *Brachypodium distachyon* [8], chicory [9], poplar [10], coffee [11], *Oenanthe javanica* (BI.) [12], and peach [13]. As of this writing, no systematic strategy is available to analyze carrot reference genes under abiotic stress and hormone stimuli conditions.

Hundreds of potential housekeeping genes have been identified by microarray analyses in *Arabidopsis* [7]. However, previous studies indicated that genes commonly used as internal controls are 3-glyceraldehyde phosphate dehydrogenase (*GAPDH*), translation elongation factor EF-1 alpha (*EF-1α*), poly-ubiquitin (*UBQ*), actin (*ACTIN*), and tubulin (*TUB*) [14–20]. These genes referred to as housekeeping control genes and played housekeeping roles in basic cellular processes, such as cell structure maintenance or primary metabolism [7], although we refer them here simply as reference genes. Currently, some new reference genes are well described for the normalization of expression signals including protein phosphatase 2A (*PP2A*), genes encoding F-box/kelch-repeat protein (*F-box*), SAND family protein (*SAND*), Eukaryotic translation initiation factor 4α (*eIF-4α*), and Tap42-inter-acting protein of 41 kDa (*TIP41*) [7,21–23]. However, several studies have scrutinized that some commonly used reference genes like *ACTIN* and *GAPDH* showed different behaviors in different plants, tissues, and experiment conditions, and these should be used with caution as internal controls [24,25]. The reason for these expressional variabilities may be that transcript levels of reference genes could vary considerably in response to experimental conditions, cellular process, and tissue types [26–28]. The normalization will produce misleading results, if the selected reference gene has a large expression fluctuation [13]. Hence, the appropriate reference genes for qPCR must be selected to obtain normalization of RNA quantitation and experimental data in different samples and to ensure the accuracy and reliability of the experimental results. Moreover, the optimal number of reference genes should be determined and multiple reference genes are required for gene expression study instead of a single gene [29].

Carrot (*Daucus carota* L.) is a biennial herb cultivated around the world and belongs to the *Daucus* genus in the Apiaceae family (Fig. A and B in S1 File). Carrot contains abundant β-carotene which imports benificial properties to human health, like anti-cancer, antioxidant, detoxification, cardiovascular protection, cataract prevention and treatment, and liver protection [30–33]. Phytohormones including salicylic acid (SA), methyl jasmonate (MeJA), gibberellic acid (GA), and abscisic acid (ABA), are known to play important roles in the regulation of plant developmental processes, and responses to biotic and abiotic stresses [34,35]. Exogenous SA could increase plant tolerance to the abiotic stress by regulating the activities of antioxidant enzymes [36]. In carrot, SA has been shown to positively affect the carotenoids and anthocyanin content, storage root dry weight, and increase the total antioxidant activity of the shoot and storage root [37]. MeJA treatment could increase the content of phytoalexin 6-methoxymellin [38]. Exogenous GA could be applied in vernalization to prevent the inhibitory effect of high temperature on seedstalk elongation [39]. Moreover, accumulation of ABA could suppresses precocious germination and modulates seed gene expression in developing seeds [40]. Environmental stresses such as drought, high salt, and temperature change could reduce productivity and significant crop losses, like drought and salinity, which together result in a more than 50% decline in the average yields of major crops worldwide [41,42]. Abiotic stresses, including heat, cold, drought, and salinity tolerance, are also known to limit carrot production [30].

In this study, nine candidate reference genes (*TIP41*, *TUB*, *eIF-4α*, *UBQ*, *SAND*, *GAPDH*, *EF-1α*, *PP2A*, and *ACTIN*) were selected based on their stable expression in previous studies [12,21,28,43,44]. The nine gene sequences of carrot were obtained based on the carrot genome sequence data, which was built by our group (Lab of Apiaceae Plant Genetics and Germplasm Enhancement, Nanjing Agricultural University) (http://apiaceae.njau.edu.cn/carrot/). Information on these reference genes is presented in Table 1. Three different algorithms (geNorm, NormFinder, and BestKeeper) were used to evaluate the expression stability of the reference genes. The experimental data of the genes were determined by qPCR in carrot leaves under different hormone stimuli treatments (GA, SA, ABA, and MeJA, respectively) and abiotic stresses treatments (heat, cold, salt, and drought). All nine reference genes displayed a wide range of quantification cycle (Cq) values across experimental samples, indicating variable expression. Furthermore, the expression level of *DcDREB-A1*, the homolog of *AtDREB-A1* (DREB, Dehydration responsive element binding factor) gene of *Arabidopsis*, was assessed using different reference genes to validate the selection of candidate reference genes. We assumed that the reference genes identified in current study would enable better normalization and quantification of transcript levels in future expression studies on carrot plants.

**Table 1:** Descriptions of reference genes in carrot (The lists of primers used in qPCR).

Gene symbol	Gene name	*Arabidopsis* homolog gene	Primer sequence (5’–3’) forward/reverse	Amplicon length (bp)	E (%)L/R	Tm(^°^C)
eIF-4α	Eukaryotic translation initiation factor 4α-1 gene	AT3G13920	TGTGCTTATCACCACTGACCTTCTG/GTCCACTACGCCCAATACGATGAA	122	108.8	82.5
ACTIN	Actin1 gene	AT2G37620	CGGTATTGTGTTGGACTCTGGTGAT/CAGCAAGGTCAAGACGGAGTATGG	98	106.2	82.5
TIP41	Tap42-interacting protein of 41 kDa gene	AT4G34270	GGAGGACTGTGAGGAACGAATTGAT/ACGCAAGAGAAGGAACCAACAACT	166	101.1	81.0
GAPDH	Glyceraldehyde-3-phosphate dehydrogenase gene	AT1G42970	AGGCTGCTGAAGGACCATTGAAG/CCATTCGTTATCGTACCAGGCTACA	164	101.2	83.5
SAND	SAND family protein gene	AT2G28390	AATGCTGCTCACTGCTAATCCAGAT/GCCACCATCCAACATCGACCTC	124	96.8	81.0
EF-1α	Elongation factor-1αgene	AT1G07940	TCAAGGATCTCAAGCGTGGTTATGT/CAGCAATGTGGCAAGTGTGACAAT	175	100.4	84.0
PP2A	Protein phosphatase 2A gene	AT4G15415	GTGTATCAATGTACCACCAGCAACT/GCTCACCAAGGAACATGACTTCTT	147	97.3	80.0
TUB	Tubulin beta-7 gene	AT2G29550	GAGTGGAGTTACCTGCTGCCTTC/ATGTAGACGAGGGAACGGAATCAAG	94	105.5	84.0
UBQ	Polyubiquitin 10 gene	AT4G05320	TCTCCGACTCCGTGGTGGTATG/CTGCCGTCCTCCAACTGCTTAC	180	93.8	85.0

## Materials and Methods

### Plant materials and treatments

Seeds of *D*. *carota* variety of Kurodagosun were sown in plastic pots containing a soil/vermiculite mixture (1:1) [45–47] and grown in an artificial climate chamber programmed for 16 h/8 h at 25°C/16°C for day/night conditions at a light intensity of ~300 μmol∙m^-2^∙s^-1^ and relative humidity 60%. Healthy and vigorous eight-week-old seedlings were used for treatments. In drought experiment, soil were irrigated with 500 mL of 20% PEG 6000 for 2 h in each pots. In salt experiment, leaves were sprayed with 500 mL of 0.2 M NaCl for 2 h. Cold and heat treatments were performed by exposing eight-week-old seedlings to 4 and 40°C temperatures in light incubators for 2 h, respectively. For hormone treatments, leaves were sprayed with 500 mL of SA (1.4 mM) [37,48], MeJA (0.8 mM) [38], GA (1.4 mM) [39], and ABA (0.1 mM) [40] for 2 h, respectively. Plants were sprayed or irrigated only once. GA, SA, MeJA (containing 0.02% (v/v) absolute ethanol and 0.02% (v/v) Tween-20), and ABA were dissolved in distilled water [49–53]. The pH of GA, SA, MeJA, and ABA dilutions were 2.8, 2.8, 6.7, and 5.3, respectively. In all cases, pots were placed in light incubators under optimal conditions with constant light intensity, being processed at the same time as plants subjected to the different stress conditions. Three biological experimental replicates were collected from three seedling samples performed in different pots for each treatment. Leaves were collected from the eight-week-old seedlings subjected to all treatments. The samples were frozen in liquid nitrogen and stored at −80°C until further use.

### Total RNA extraction and cDNA synthesis

Frozen carrot tissues were disrupted under liquid nitrogen conditions using mortar and pestle. Total RNA extraction was performed according to the manufacturer’s protocol (Tiangen, Beijing, China). The concentration and purity of RNA samples were measured by NanoDrop ND1000 spectrophotometer, and cDNA synthesis was performed using an A_260_/A_280_ ratio of 1.8 to 2.0 samples. The genetic integrity was evaluated by 1.5% agarose gel electrophoresis. cDNA was synthesized from approximately 1,000 ng total RNA using the PrimeScript RT reagent Kit with gDNA Eraser (TaKaRa, Dalian, China). The cDNA was ten-fold diluted series (10×, 10^2^×, 10^3^×, 10^4^×,10^5^×, and 10^6^× dilutions) for determining the amplification efficiency (E) and correlation coefficient (R^2^) analysis; and eighteen-fold diluted for conducting the qPCR analysis of elicitor treatments.

### Selection of candidate reference genes and primer design

Nine genes, *TIP41*, *TUB*, *eIF-4α*, *UBQ*, *SAND*, *GAPDH*, *EF-1α*, *PP2A*, and *ACTIN*, were used to identify the most stable reference genes for qPCR expression analyses of target carrot genes. These genes have already been identified and have been commonly used as internal controls in previous studies [12,21,28,44]. For this study, the *Arabidopsis* genes were selected from the TAIR database (http://www.arabidopsis.org). Potential homologs of the nine reference genes were identified from the genome and transcriptome data sequences of carrot, which were sequenced and analysized by our group (CarrotDB: http://apiaceae.njau.edu.cn/carrot/) [54]. The potential homologs sequences were aligned and edited by using BioEdit Sequence Alignment v 7.0.9 software. Primers were designed using Primer 6.0 (Premier Biosoft International, Palo Alto, CA) and DNAMAN 6.0 (Lynnon Biosoft, USA) according to the manufacturer’s instructions. The primers used in qPCR, as well as their melting temperatures (80°C to 85°C), primer lengths (22 bp to 25 bp), GC content (44% to 60%), and amplicon lengths (80 bp to 180 bp) are provided in Table 1. Cloning information is presented in Table A in S1 File. The specificity of the amplicons was verified by using a single band of expected size in 1.5% agarose gel following electrophoresis and by the presence of a single peak in the qPCR melting curve. The target amplicons were sequenced to confirm specificity of the PCR products.

### Quantitative real-time PCR assay

The qPCR was designed according to the minimum information for publication of quantitative real-time PCR experiment guidelines [55]. Reactions used SYBR Green I Mix (TaKaRa, Dalian, China) in a 20 μL reaction volume and were performed in a 96-well plate on MyiQ single color real-time PCR detection system (Bio-Rad, Hercules, USA). Reaction mixtures contained 10 μL SYBR Green I Mix, 2 μL diluted cDNA, ddH_2_O, and a final primer concentration of 0.4 μM. The following amplification conditions were applied: an initial denaturation step of 95°C for 30 s; 40 cycles at 95°C for 5 s; and 60°C for 20 s. The final dissociation curve was obtained from 65°C to 95°C to verify primer specificity. Each assay included three technical and biological replicates, and a standard curve of six serial dilution points. The general quality assessment of the PCR results was based on the amplification and melting curve profiles of the samples in relation to the assay controls (non-template controls). Mean Cq values of the ten-fold dilution series were plotted against the logarithm of the pooled cDNA dilution factors. The Cq values and the following equation were used to determine efficiency (E) of each gene with the slope of a linear regression model: % E = (10^[−1/slope]^ - 1) × 100% [56]. Amplification efficiencies were calculated from standard curves with satisfactory linear relationships (R^2^ > 0.99). All PCR processes displayed efficiency between 90% and 110%.

### Data analysis

Three different types of Microsoft Excel-based software, namely, geNorm [29], NormFinder [57], and BestKeeper [58], were used to rank the expression stability of reference genes across all experimental sets. These data were either used directly for stability calculations (BestKeeper analysis) or were converted into relative quantities and imported into the geNorm and NormFinder using the formula 2^-ΔCq^, in which ΔCq = the corresponding Cq value—minimum Cq. The raw data are listed in Table B in S1 File.

In geNorm, the reference gene expression stability measurement (M) value is calculated as the level of pairwise variation for each reference gene with all other control genes and as the standard deviation (SD) of the logarithmically transformed expression ratios [29]. The reference gene with the lowest M value is considered the most stable gene [59]. Similar to geNorm, the NormFinder program is another Visual Basic application tool for Microsoft Excel that is used to determine the expression stabilities of reference genes [12]. Misinterpretations caused by artificial selection of co-regulated genes are avoided with this program [57]. BestKeeper determines the most stably expressed genes based on the coefficient of correlation to the candidate reference gene’s Cq values [58]. Genes with the lowest SD and CV values are the most stable [60].

## Results

### Cq values of candidate reference genes in carrot

**Fig 1:**
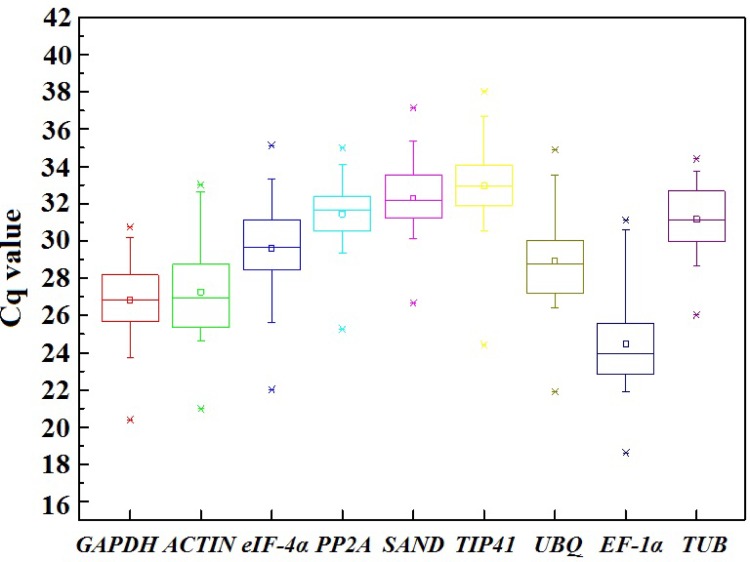
Cq values of candidate reference genes in all carrot samples. Asterisks denote outliers. The line across the box depicts the median value. The inside box depicts Cq values. The outside box’s bottom line is determined by the 25^th^ percentile, whereas the top line is determined by the 75^th^ percentile. The top and bottom whiskers are determined by the 5^th^ and 95^th^ percentiles, respectively.

**Fig 2:**
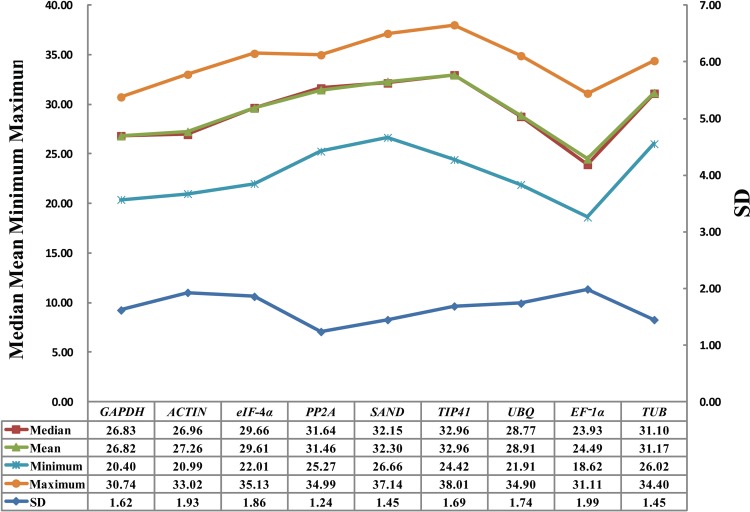
Data statistics of Cq values of candidate reference genes in carrot. Total number of Cq values in each reference genes is 72. Mean, median, minimum, and maximun of Cq values were determined by statistic analysis. SD of the Cq values were generated by BestKeeper.

Based on primer sequences from Table A in S1 File, cDNA of nine genes were cloned and identified in carrot leaves based on the data of carrot genome and transcriptome sequences. The gene expression levels were determined as Cq values (Table B in S1 File), and the transcripts of the reference genes showed different levels of abundance (Fig. 1). Mean Cq values of the genes ranged from 24.49 (*EF-1α*) to 32.96 (*TIP41*), and the Cq values of all the tested samples were between 18.62 (*EF-1α*) and 38.01 (*TIP41*). Low Cq values corresponded to high levels of expression. *EF-1α* showed high expression level with low Cq value. *TIP41* and *SAND* showed low expression levels with high Cq values (Fig. 2).

### Determination of the optimal number of reference genes in carrot

**Fig 3:**
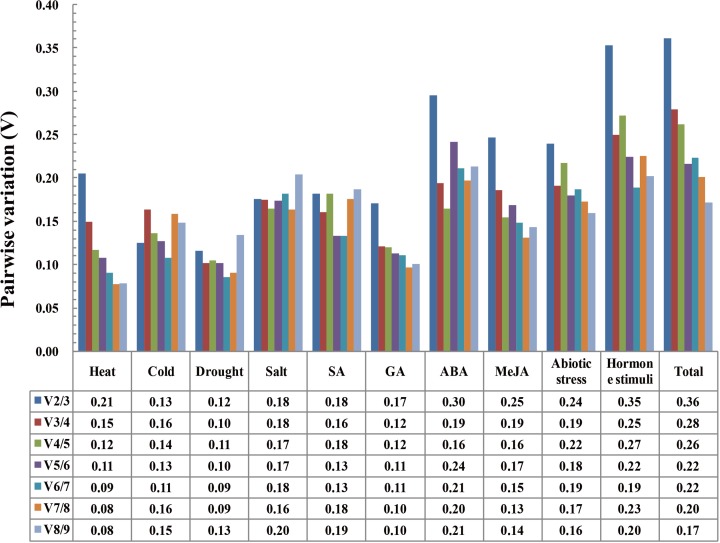
Determination of the optimal number of reference genes. Pairwise variation (V_n/n +1_) analysis between the normalization factors (NF_n_ and NF_n +1_) was performed by using the geNorm program in all samples MeJA, methyl jasmonate; SA, salicylic acid; GA, gibberellin; and ABA, abscisic acid. The abiotic stress group included heat, cold, drought, and salt treatments. The hormone stimuli group include SA, GA, ABA, and MeJA. The total group included all samples.

The optimal number of reference genes required for normalization was determined with geNorm using pairwise variations (Vn/n + 1) between the sequential normalization factors (NFn and NFn + 1, n ≥ 2). A large variation between the sequential normalization factors indicates that the added gene has a significant effect and is preferred for inclusion and calculation of a reliable normalization factor [29]. As shown in Fig. 3, the third gene had no significant effect (V_2/3_, low value) in cold and drought conditions. Thus, two reference genes were sufficient for normalizing gene expression under the cold and drought conditions. With a threshold of 0.15, three genes were sufficient for normalizing gene expression under heat and GA stress conditions, five for SA stress and six for MeJA stress. None of the gene selected was found to be appropriate in salt stress condition in the current study.

### Expression stability of candidate reference genes in carrot

Three different software programs were used to calculate the expression stability of the candidate reference genes: geNorm, NormFinder, and BestKeeper. Eight different treatment sets were sorted into three groups: “abiotic stress” (heat, cold, salt, and drought), “hormone stimuli” (SA, GA, ABA, and MeJA), and “total” (samples in all treatments). Accordingly, 11 evaluation patterns were generated for both single stress treatments and groups.

According to geNorm, in which the default limit comprised M values less than 1.5, and except for *TIP41* under ABA stress, all the other reference genes performed well under individual stress conditions (Table C in S1 File). *EF-1α* and *ACTIN* were the two best genes among the nine reference genes in SA and salt stress treatments. However, in the MeJA treatment, *EF-1α* and *UBQ* were the two best reference genes. In NormFinder, *TIP41* was the most stable gene among the nine candidate genes under salt and SA stress conditions. *UBQ* was the most stable gene under GA and cold stress conditions. The BestKeeper analysis showed that most of the nine candidate genes had satisfactory stability. *TUB*, *GAPDH*, and *UBQ* were ranked at the top positions in most of the single stress treatments by BestKeeper analysis. All candidate genes were confirmed to be stable under GA treatment in BestKeeper.

Recognizing the best reference gene was difficult because of the complexity of the groups. The results of the analysis of the three groups of samples are shown in Table 2. The nine candidate genes performed well by geNorm analysis. In the “abiotic stress” group, *ACTIN* and *UBQ* were the two most stable genes, and *eIF-4α* and *GAPDH* ranked top two in the “hormone stimuli” group. *ACTIN* and *EF-1α* were the two most stable genes in the “total” group. *ACTIN*, *EF-1α*, and *GAPDH* performed well in all three groups by geNorm analysis. *eIF-4α* was the most stable reference gene with the minimum value of 0.005 obtained by NormFinder in the “hormone stimuli” group. *ACTIN* was the most stable reference gene with the value of 0.012 and 0.015 obtained by NormFinder in “total” and “abiotic stress” groups, whereas it ranked fourth in “hormone stimuli” group. *GAPDH* performed well in “hormone stimuli” group by NormFinder analysis, while it ranked the last one in “abiotic stress” and “total” group. In all three groups, *ACTIN*, *eIF-4α*, and *TIP41* performed well in terms of stability according to NormFinder analysis. In BestKeeper, *EF-1α* was the most stable reference gene in “abiotic stress” group, whereas it was the least stable in “hormone stimuli” and “total” groups; *PP2A* ranked first in “hormone stimuli” and “total” group and it ranked fifth in “abiotic stress” group. *TUB* and *GAPDH* were more stable than the other genes in all three groups according to BestKeeper. In both “abiotic stress” and “total” groups, *ACTIN* was ranked first according to geNorm and NormFinder; whereas *ACTIN* was ranked sixth and eighth by BestKeeper, respectively.

**Table 2:** Gene expression stability in carrot under multiple stress treatments, as ranked by the three software programs geNorm, NormFinder, and BestKeeper.

Group	Rank	geNorm		NormFinder		BestKeeper		
		Gene	Stability	Gene	Stability	Gene	SD	CV
Abiotic stress	1	ACTIN	0.68		ACTIN	0.015	EF-1α	1.06	4.63
	2	UBQ	0.68	TIP41	0.015	GAPDH	1.33	5.16
	3	EF-1α	0.76	PP2A	0.029	TUB	1.33	4.41
	4	GAPDH	0.82	eIF-4α	0.029	UBQ	1.33	4.84
	5	PP2A	0.96	SAND	0.039	PP2A	1.36	4.38
	6	TUB	1.06	TUB	0.041	ACTIN	1.37	5.32
	7	SAND	1.18	EF-1α	0.058	SAND	1.45	4.59
	8	eIF-4α	1.28	UBQ	0.060	TIP41	2.00	6.19
	9	TIP41	1.36	GAPDH	0.068	eIF-4α	2.25	7.84
Hormone stimuli	1	GAPDH	0.98	eIF-4α	0.005	PP2A	1.03	3.23
	2	eIF-4α	0.98	GAPDH	0.006	SAND	1.07	3.24
	3	ACTIN	1.10	TIP41	0.007	TUB	1.14	3.56
	4	EF-1α	1.14	ACTIN	0.007	TIP41	1.37	4.09
	5	TUB	1.29	UBQ	0.009	GAPDH	1.41	5.05
	6	TIP41	1.39	EF-1α	0.011	eIF-4α	1.58	5.17
	7	UBQ	1.45	PP2A	0.012	UBQ	1.59	5.26
	8	SAND	1.59	TUB	0.015	ACTIN	1.83	6.37
	9	PP2A	1.70	SAND	0.017	EF-1α	2.05	7.84
Total	1	ACTIN	0.85	ACTIN	0.012	PP2A	1.24	3.95
	2	EF-1α	0.85	TIP41	0.014	TUB	1.45	4.64
	3	GAPDH	1.06	eIF-4α	0.021	SAND	1.45	4.49
	4	UBQ	1.16	PP2A	0.022	GAPDH	1.62	6.04
	5	TUB	1.29	SAND	0.029	TIP41	1.69	5.13
	6	eIF-4α	1.37	TUB	0.033	UBQ	1.74	6.02
	7	TIP41	1.49	UBQ	0.044	eIF-4α	1.86	6.27
	8	SAND	1.59	EF-1α	0.044	ACTIN	1.93	7.10
	9	PP2A	1.65	GAPDH	0.048	EF-1α	1.99	8.12

The reference gene stability in carrot was analyzed. Three groups were formed: abiotic stress group (heat, cold, salt, and drought); hormone stimuli (SA, GA, MeJA, and ABA); and total (all samples).

### Reference gene validation

**Fig 4:**
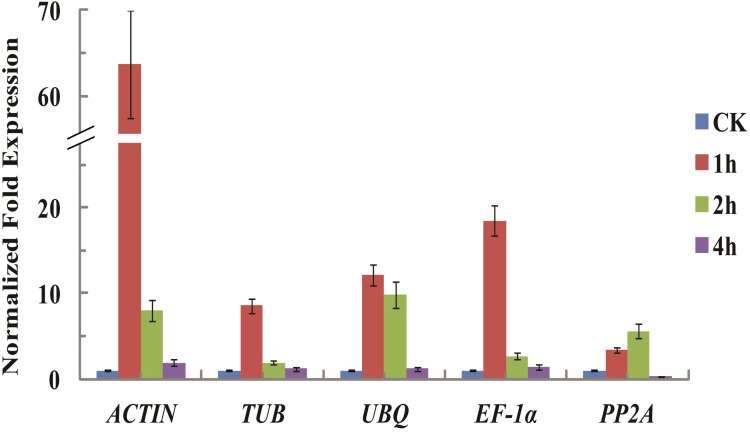
Relative quantification of *DcDREB-A1* gene expression normalized using candidate reference genes under heat treatment in carrot.

To validate the selection of candidate reference genes, the relative expression of *DcDREB-A1* was calculated by using the selected reference genes (Fig. 4). In *A*. *thaliana*, the expression of the *AtDREB-A1* gene was induced by abiotic stress treatments, including cold, drought, and heat [61–63]. In this study, the expression profiles of *DcDREB-A1* in carrot under heat stress condition were assessed by using six candidate reference genes. When the two most stable reference genes *ACTIN* and *TUB* were used for normalization, the expression levels of *DcDREB-A1* peaked at 1 h and subsequently decreased at 2 and 4 h (Fig. 4). When the less stable reference gene *UBQ* and *EF-1α* were used for normalization, similar expression patterns were generated. By contrast, when *PP2A* were used for normalization, the transcript levels and expression patterns differed from those obtained using *ACTIN* and other suitable reference genes. When normalization was conducted based on the least stable reference gene *PP2A*, the expression patterns of *DcDREB-A1* peaked at 2 h and decreased at 4 h.

## Discussion

qPCR is broadly accepted as a method with high sensitivity and specificity. Such method is used because of its repeated quantitative dynamic range and the high-throughput analysis of gene transcript levels. Accurate normalization of gene expression against an appropriate internal control is required for a valid qPCR analysis. Gene transcripts with invariant abundance under various environmental stimuli are essential reference points for accurate data analysis [7]. Thus, reference genes should be validated under certain experimental conditions and in different species [60,64].

Leaves serve important functions in process of photosynthesis. In the growth and development of carrot, the accumulated photosynthetic products were transported to tuberous roots. Furthermore, leaf is a vital organ of the response to the abiotic stress and hormone signal. In this study, we tested suitable reference genes for the expression of target genes in carrot leaves. Nine genes (*GAPDH*, *ACTIN*, *eIF-4α*, *PP2A*, *SAND*, *TIP41*, *UBQ*, *EF-1α*, and *TUB*) were selected as candidate reference genes for stable expression assessment tests in carrot leaves. All nine candidate genes were cloned from carrot in this study based on our transcriptome and genome database, CarrotDB [54]. A single peak in the melting curve analyses confirmed the primer pair that showed specificity. Plants were subjected to different hormone stimuli (GA, SA, MeJA, and ABA), abiotic stresses (heat, cold, salt, and drought), and efficacy dilutions (10×, 10^2^×, 10^3^×, 10^4^×,10^5^×, and10^6^× dilutions). The expression data were collected following qPCR amplification and detection. The curves showed a good linear relationship default limit with R^2^ > 0.99, and their amplification efficiencies ranged from 93.8% to 108.8% (Table 1). The primer pairs and amplification conditions were acceptable in qPCR-based quantification [55].

Most of the Cq values were lying between 18 and 35 across all tested samples, and the mean Cq values ranged from 24 to 33 [12,60,65,66]. Here, the Cq values of *PP2A* (Cq_max_—Cq_min_ <10 cycles; SDs = 1.24) were distributed more centrally than those of the other candidate genes, whereas the Cq value of *EF-1α* showed the highest variation (Cq_max_—Cq_min_ <13 cycles; SD = 1.99). *TUB*, *eIF-4α*, *ACTIN*, *PP2A*, *GAPDH*, and *UBQ* showed moderate expression levels. The mean values of all reference genes except *EF-1α* were close to the median values of these candidate genes, indicating that Cq values are evenly distributed.

We compared three different approaches implemented in the software programs, namely, geNorm, NormFinder, and BestKeeper. Each program differed in terms of the composition and ranking of the most stably expressed reference genes candidates; such differences may be caused by the variations between the approaches [3]. Comparison of the results obtained from the three software programs could reveal the most stable reference genes under specific experimental conditions. We also have determined the optimal number of reference genes required for accurate normalization. However, setting a cut-off value was used in some references but not a necessary criterion [29].

In heat stress, three genes were sufficient for normalizing gene expression under heat stress conditions calculated by geNorm (V_3/4_ value = 0.15). The software program suggested the use of *eIF-4α*, *SAND* and *TUB* for normalization. *SAND* was ranked the best reference gene in NormFinder, while eighth in BestKeeper. *EIF-4α* was ranked fourth in NormFinder, while seventh in BestKeeper. *TUB* was ranked fifth in BestKeeper, sixth in NormFinder. *EF-1a* was ranked the best reference gene in BestKeeper, sixth in geNorm and the last one in NormFinder. *ACTIN* was ranked the third in BestKeeper, fourth in geNorm and fifth in NormFinder. We considered the rankings of three algorithms together, and recommended *ACTIN* and *TUB* combined with *eIF-4α* or *SAND*, as the best combination of stable reference genes for qPCR in the heat treatment.

In cold stress, the pairwise variation V_2/3_ = 0.13, indicated that the addition of third gene had no significant effect for normalization. Two most stable genes *ACTIN* and *UBQ* can be used by geNorm analysis. *ACTIN* could be the preferred reference gene with the ranking of fifth and sixth by NormFinder and BestKeeper. *UBQ* was identified by NormFinder as the most stable reference gene and showed a variation in BestKeeper (seventh-ranked). BestKeeper ranked *SAND* as most stable, while ranked seventh by NormFinder and eighth by geNorm. BestKeeper ranked *GAPDH* at the fifth position and it was ranked fourth by geNorm and NormFinder. Based on these results, *UBQ* combined with *ACTIN* or *GAPDH* were recommended as the best combination of stable reference genes for normalization in cold treatment.

In drought stress, the pairwise variation V_2/3_ = 0.12, indicated that two genes were sufficient for normalizing gene expression according to geNorm. Two most stable genes *GAPDH* and *ACTIN* could be used in qPCR by geNorm analysis. *GAPDH* could be the preferred reference gene with the ranking of third and ninth by BestKeeper and NormFinder, respectively. *ACTIN* was identified by NormFinder as the most stable reference gene and ranked the second place in BestKeeper. BestKeeper ranked *TUB* as most stable, and third-ranked by NormFinder, while ranked eighth by geNorm. Based on these results, *ACTIN* combined with *TUB* or *GAPDH* were recommended as the suitable combination of stable reference genes for normalization in drought treatment. Similarly, *GAPDH* combined with *eIF-4α* and *UBQ* would be sufficient for the GA treatment; the suitable combination of *GAPDH*, *ACTIN*, *eIF-4α*, *TIP41*, and *EF-1α* would be sufficient for the SA treatments, and a suitable combination of *GAPDH*, *eIF-4α*, *PP2A*, *SAND*, *UBQ*, and *EF-1α* would be sufficient for the MeJA treatment.

The results of BestKeeper analysis in the three groups, namely, “abiotic stress”, “hormone stimuli”, and “total”, indicated that they did not perform well. In “abiotic stress” group, two most stable genes *ACTIN* and *UBQ* can be used in qPCR by geNorm analysis. *ACTIN* was identified by NormFinder as the most stable reference gene and ranked sixth in BestKeeper; *UBQ* could be the preferred reference gene with the ranking of second by geNorm and BestKeeper and eighth calculated by NormFinder; *EF-1a* was identified by BestKeeper as the most stable reference gene and ranked third and seventh in geNorm and NormFinder, respectively; BestKeeper ranked *TUB* at the second place (SD of *TUB*, *UBQ* and *GAPDH* = 1.33), and sixth-ranked by NormFinder and geNorm. *ACTIN*, *UBQ*, *EF-1a* and *TUB* were chosen as the stable reference gene combination in “abiotic stress” group. Similarly, *eIF-4α*, *GAPDH*, *ACTIN*, and *TUB* were selected for “hormone stimuli” group; *ACTIN* and *TUB* could be chosen as reference genes for the “total” group.

In recent studies, *UBQ* showed stability in tomato [67] and *A*. *thaliana* [7], however, failed to perform satisfactorily in rice [59] and soybean [68]. *GAPDH* is among the best reference genes for measuring gene expression in many tissues [11,19,69]. *ACTIN* showed instability under numerous experimental conditions [70], but this gene is shown to be a suitable reference gene in developmental studies [68]. *TUB* also displayed a acceptably variable expression pattern and could be regarded as a commonly used reference gene in recent studies [7,60,66].

Plants respond to abiotic stress in their environments in developmental, physiological, and biochemical ways using a network of transcription factors [71,72]. AP2/ERF transcription factor (APETALA2/ethylene-responsive factor) is a large family of plant-specific transcription factors that activates the expression of abiotic stress-responsive genes *via* specific binding to the dehydration-responsive element and *cis*-acting element in their promoters [73–75]. These DREB homolog genes were induced by heat in many plants, for example *Zea mays* [76], Chinese cabbage [77], *Arabidopsis thaliana* [78], and so on. Previous studies have shown that heat shock transcription factors could be transcriptionally controlled by DREB and important for the establishment of thermotolerance [78,79]. Over-expression of several DREB transcription factors in transgenic plants could enhance tolerance to heat stress in plants [42]. We collected six reference genes to normalize the relative expression of *DcDREB-A1* under heat stress condition. The expression level of the *DcDREB-A1* gene was normalized by the most stable reference genes (*ACTIN* and *TUB*). The less stable reference genes (*UBQ* and *EF-1α*) showed similar expression patterns, but expression levels varied for these reference genes. When *PP2A* was used for normalization, the expression patterns and transcript levels obviously differed from those obtained by normalization against *ACTIN* and other suitable reference genes. Thus, use of an untested reference gene may reduce accuracy or produce misleading results.

To our knowledge, this study is the first systematic analysis for the selection of superior reference genes for qPCR in carrot leaves under different ‘abiotic stress’ (osmotic, salt, cold and heat), ‘Hormone stimuli’ (SA, GA, ABA, and MeJA), and ‘Total’ (samples in all treatments) conditions. The most stable reference gene were not the same ones depending on the stress. This study also proved that no single gene could express stably in all cell types and under all experimental conditions. Our shortlist may provide further supports to find putative candidate genes for future experiments that address other environmental variables as treatment factors in carrot.

## Supporting Information

S1 File Contains Figs. A-L and Tables A-C. **Fig. A.** Photograph of plants of *D*. *carota* variety of five-inche Kuroda. **Fig. B.** Photograph of plants of *D*. *carota* variety of five-inche Kuroda. **Fig. C.** Nucleotide acid and deduced amino acid sequences of GAPDH from carrot. **Fig. D.** Nucleotide acid and deduced amino acid sequences of ACTIN from carrot. **Fig. E.** Nucleotide acid and deduced amino acid sequences of eIF-4α from carrot. **Fig. F.** Nucleotide acid and deduced amino acid sequences of PP2A from carrot. **Fig. G.** Nucleotide acid and deduced amino acid sequences of SAND from carrot. **Fig. H.** Nucleotide acid and deduced amino acid sequences of TIP41 from carrot. **Fig. I.** Nucleotide acid and deduced amino acid sequences of UBQ from carrot. **Fig. J**. Nucleotide acid and deduced amino acid sequences of EF-1α from carrot. **Fig. K**. Nucleotide acid and deduced amino acid sequences of TUB from carrot. **Fig. L.** Standard curves of each candidate genes. **Table A.** Primer sequences for clone of nine reference genes from carrot. **Table B.** Raw Cq values in carrot. **Table C.** Gene expression stability in carrot under individual stress conditions.
(DOCX)
