# Supplementary material for: Selection of Suitable Reference Genes for qPCR Normalization under Abiotic Stresses and Hormone Stimuli in Carrot Leaves
Source: PLoS One. 2015 Feb 6;10(2):e0117569. doi: 10.1371/journal.pone.0117569 (PMC4319972; doi:10.1371/journal.pone.0117569)
Supplement: S1 File — Fig. A. Photograph of plants of D. carota variety of five-inche Kuroda. Fig. B. Photograph of plants of D. carota variety of five-inche Kuroda. Fig. C. Nucleotide acid and deduced amino acid sequences of GAPDH from carrot. Fig. D. Nucleotide acid and deduced amino acid sequences of ACTIN from carrot. Fig. E. Nucleotide acid and deduced amino acid sequences of eIF-4α from carrot. Fig. F. Nucleotide acid and deduced amino acid sequences of PP2A from carrot. Fig. G. Nucleotide acid and deduced amino acid sequences of SAND from carrot. Fig. H. Nucleotide acid and deduced amino acid sequences of TIP41 from carrot. Fig. I. Nucleotide acid and deduced amino acid sequences of UBQ from carrot. Fig. J. Nucleotide acid and deduced amino acid sequences of EF-1α from carrot. Fig. K. Nucleotide acid and deduced amino acid sequences of TUB from carrot. Fig. L. Standard curves of each candidate genes. Table A. Primer sequences for clone of nine reference genes from carrot. Table B. Raw Cq values in carrot. Table C. Gene expression stability in carrot under individual stress conditions. (DOCX) [file pone.0117569.s001.docx]

**Supporting Information**

**Fig. A.**

Photograph of plants of *D. carota* variety of five-inche Kuroda


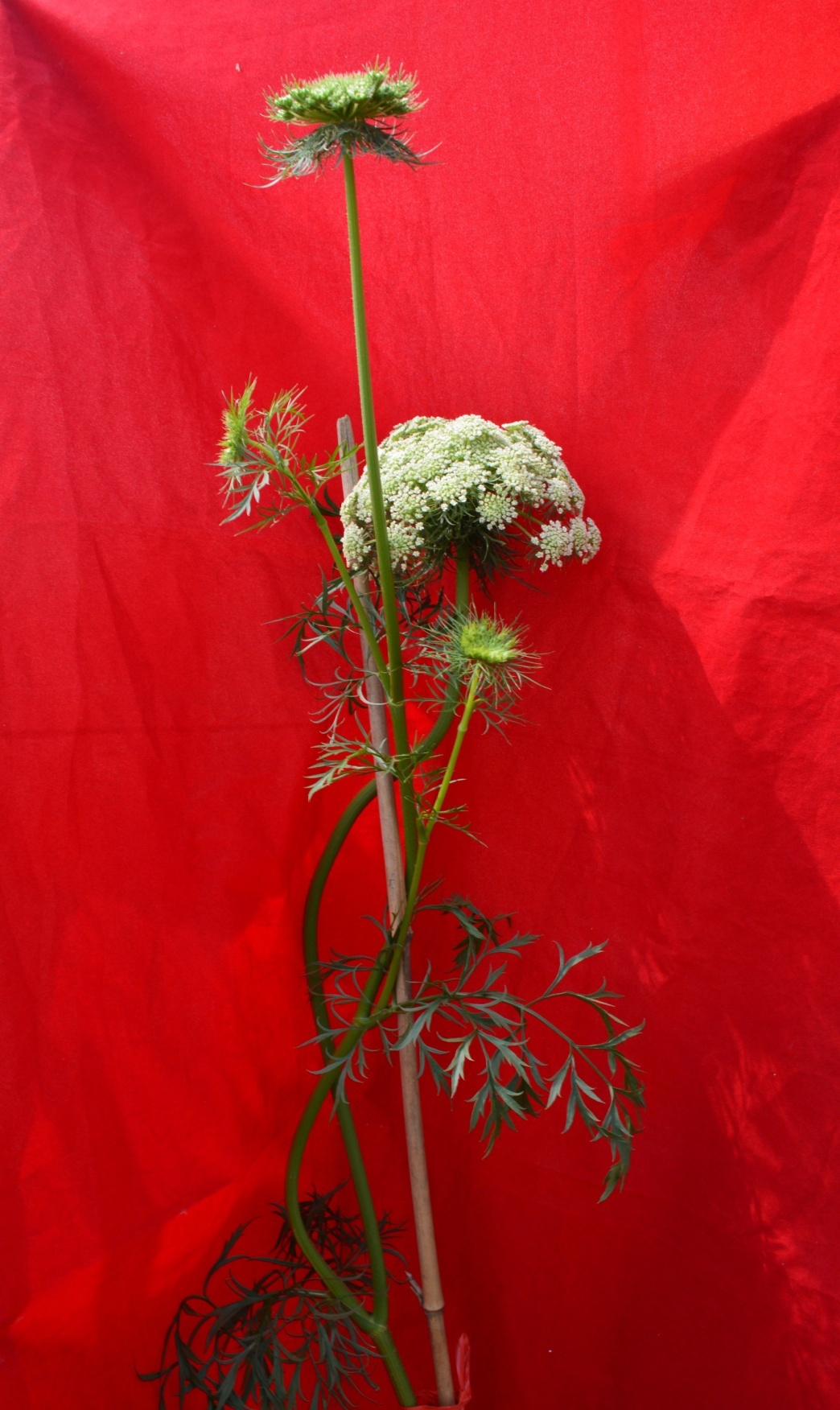


**Fig. B.**

Photograph of plants of *D. carota* variety of five-inche Kuroda (Roots)

**
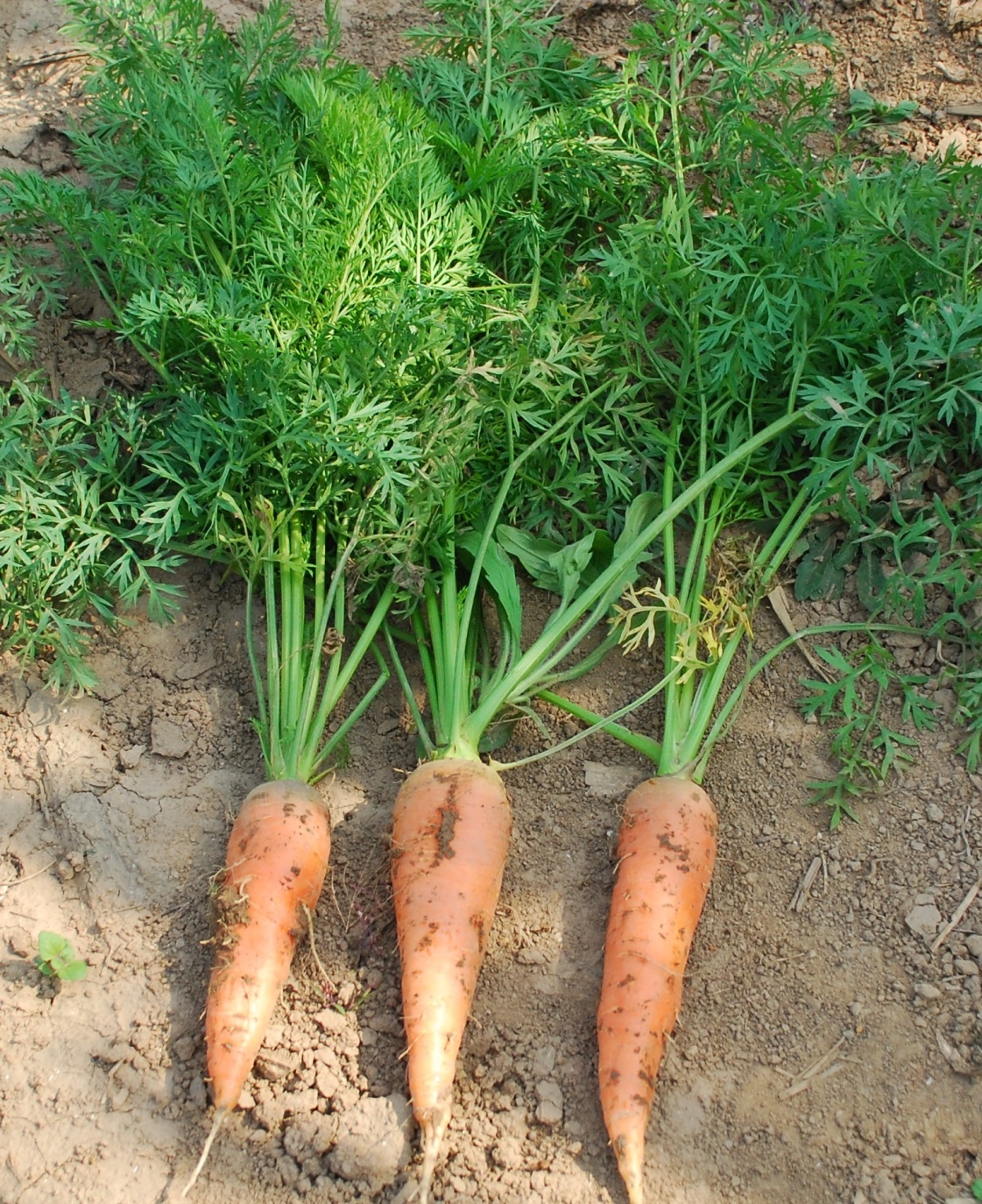
**

**Fig. C.**

Nucleotide acid and deduced amino acid sequences of *GAPDH* from carrot.

 
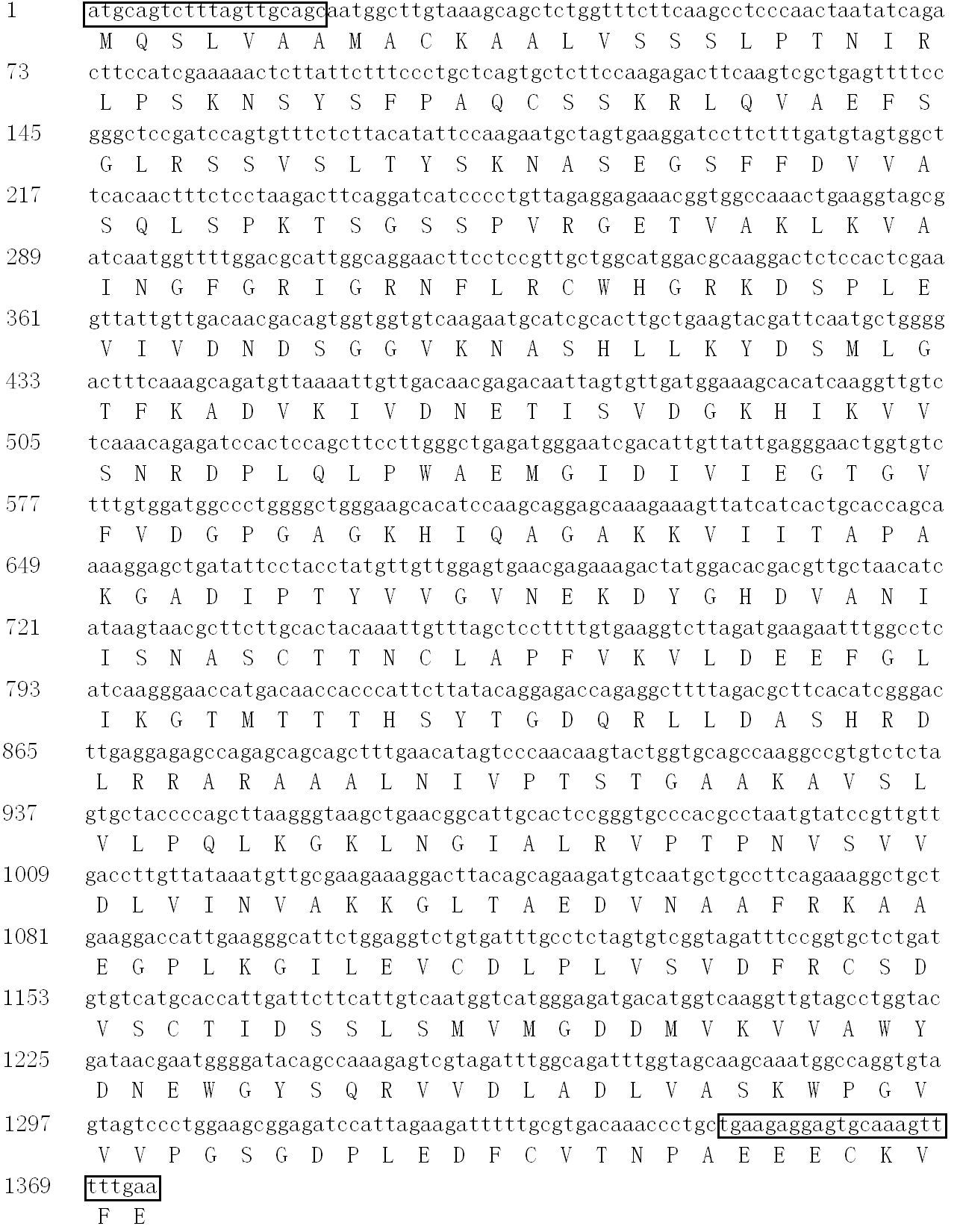


**Fig. D.**

Nucleotide acid and deduced amino acid sequences of *ACTIN* from carrot.


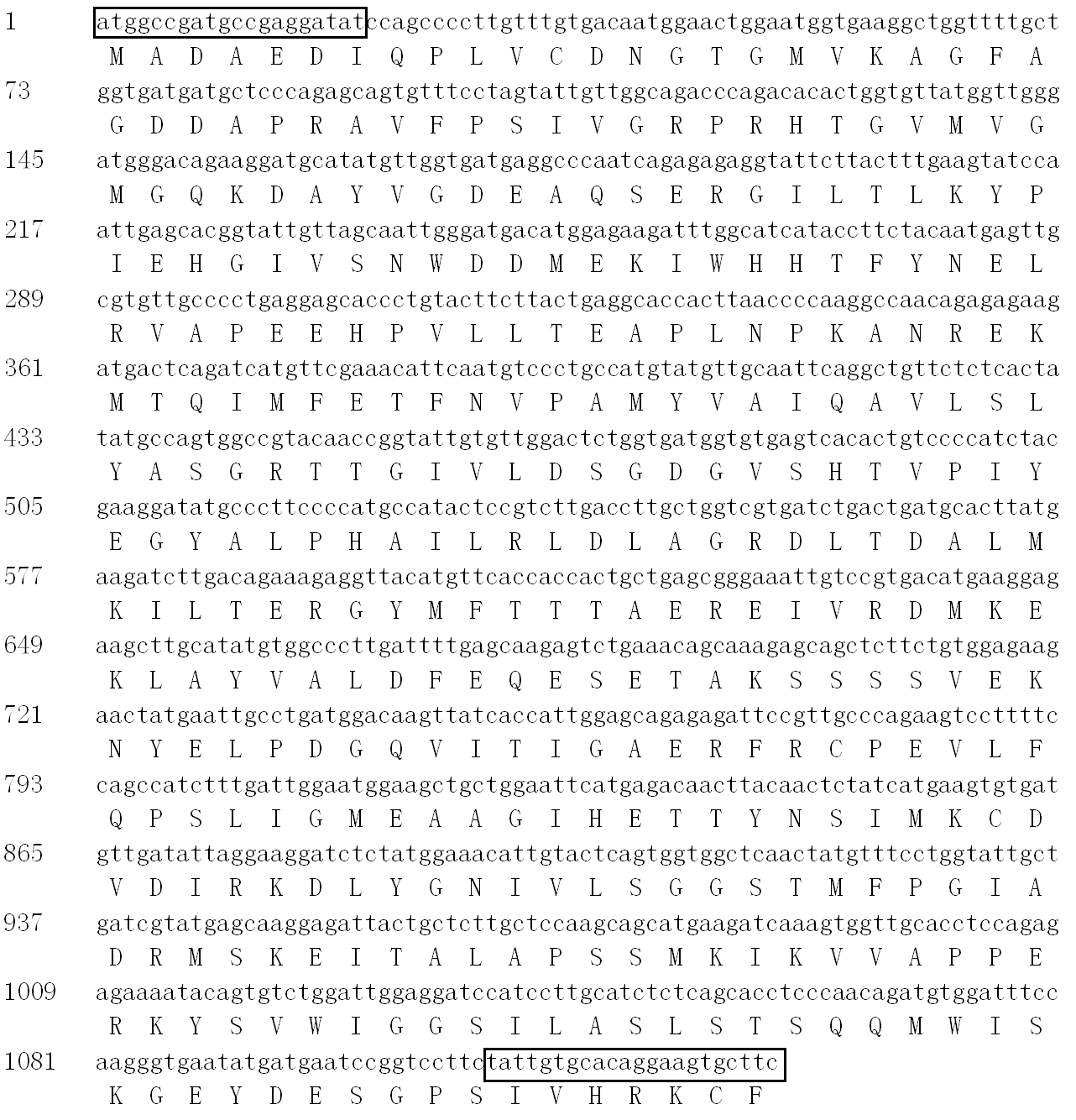


**Fig. E.**

Nucleotide acid and deduced amino acid sequences of *eIF-4α* from carrot.


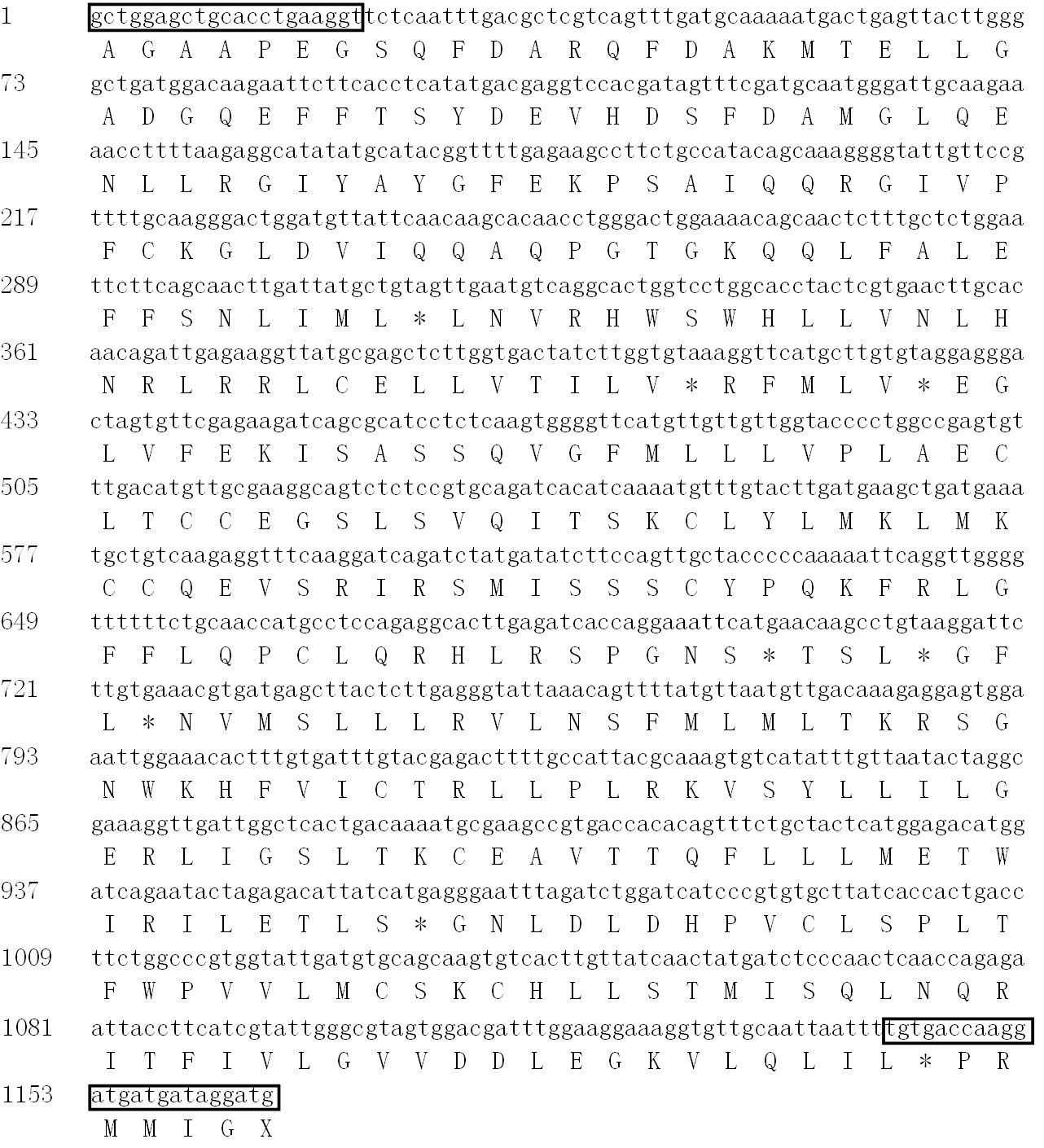


**Fig. F.**

Nucleotide acid and deduced amino acid sequences of *PP2A* from carrot.


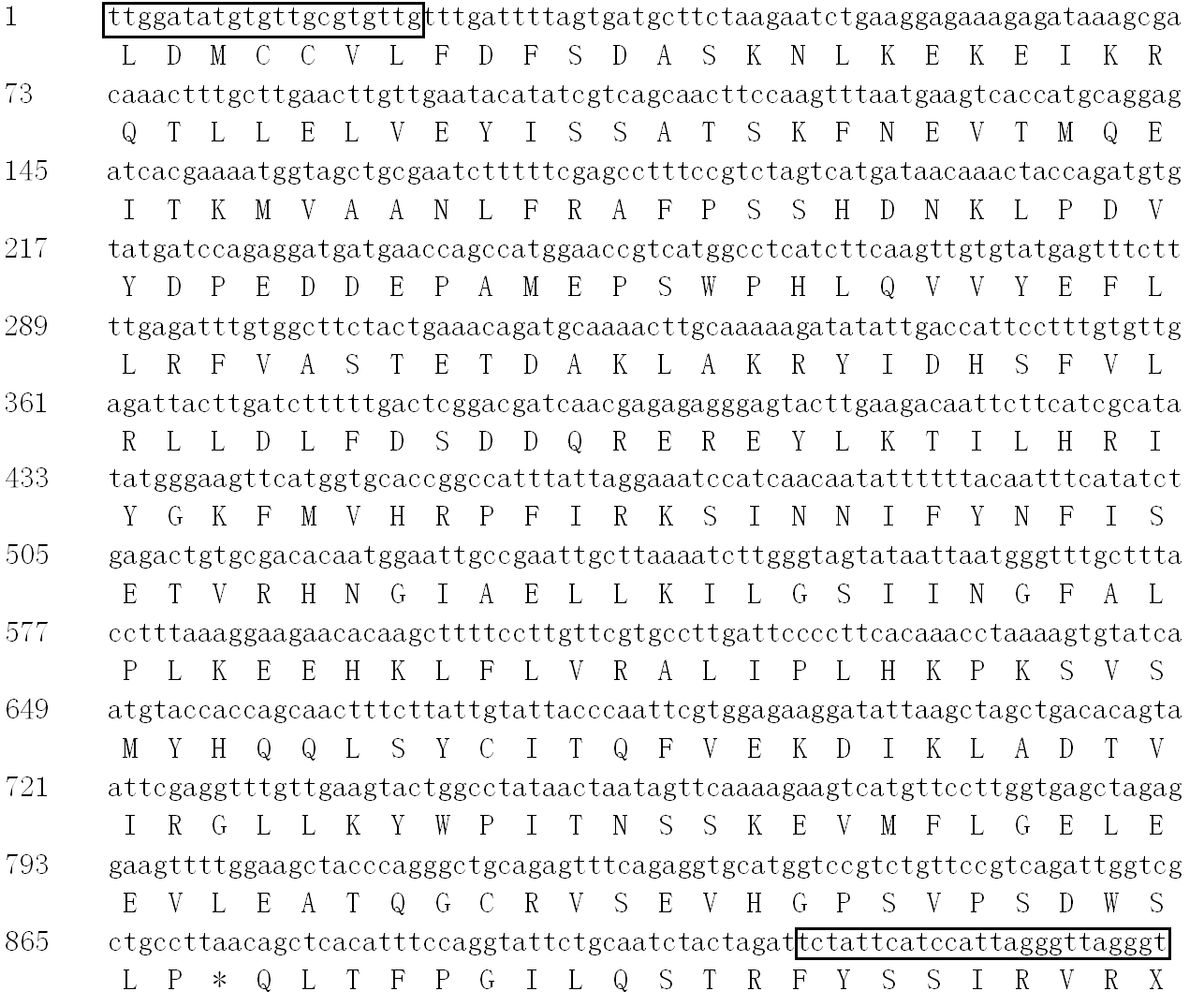


**Fig. G.**

Nucleotide acid and deduced amino acid sequences of *SAND* from carrot.


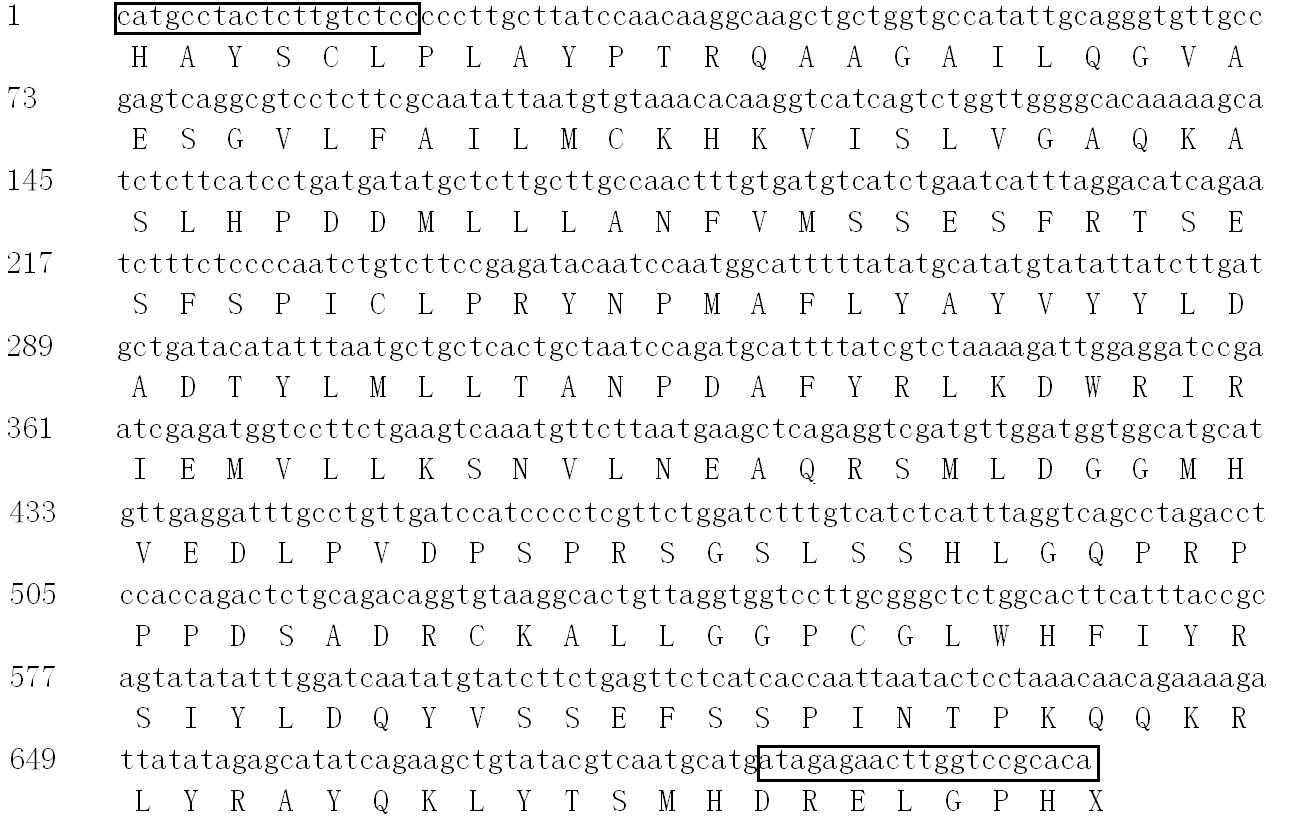


Fig. H.

Nucleotide acid and deduced amino acid sequences of *TIP41* from carrot.


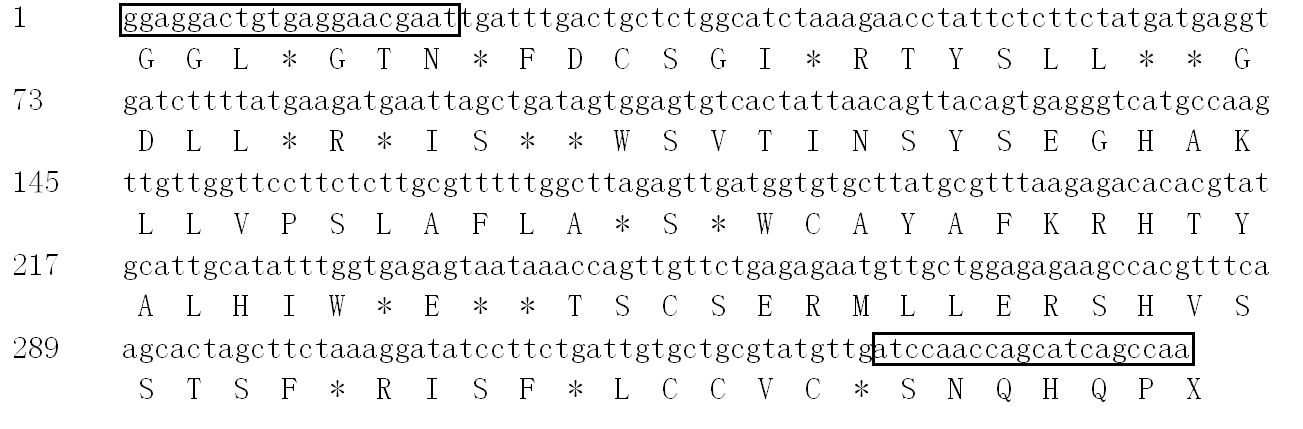


**Fig. I.**

Nucleotide acid and deduced amino acid sequences of *UBQ* from carrot.


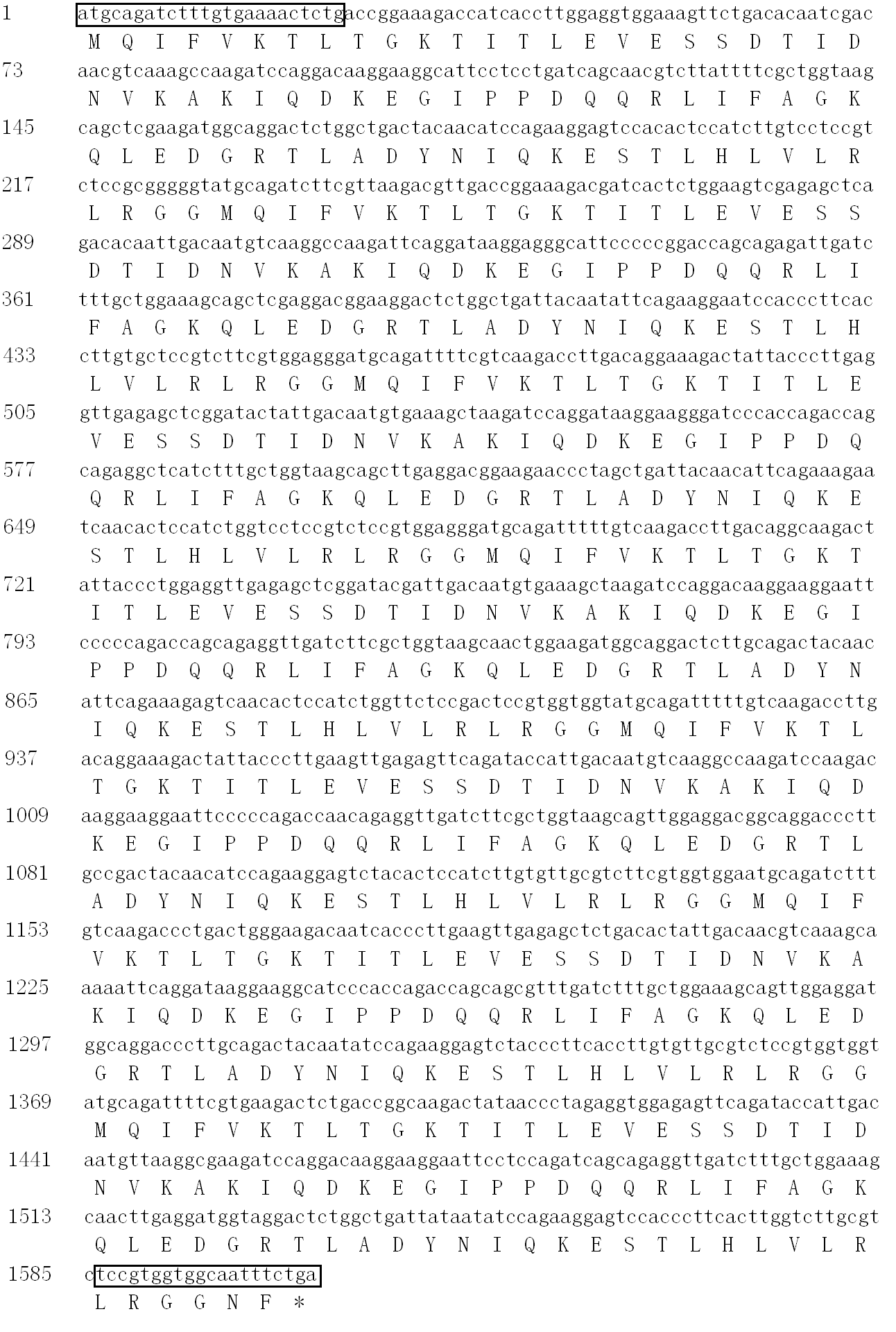


Fig. J.

Nucleotide acid and deduced amino acid sequences of *EF-1α* from carrot.


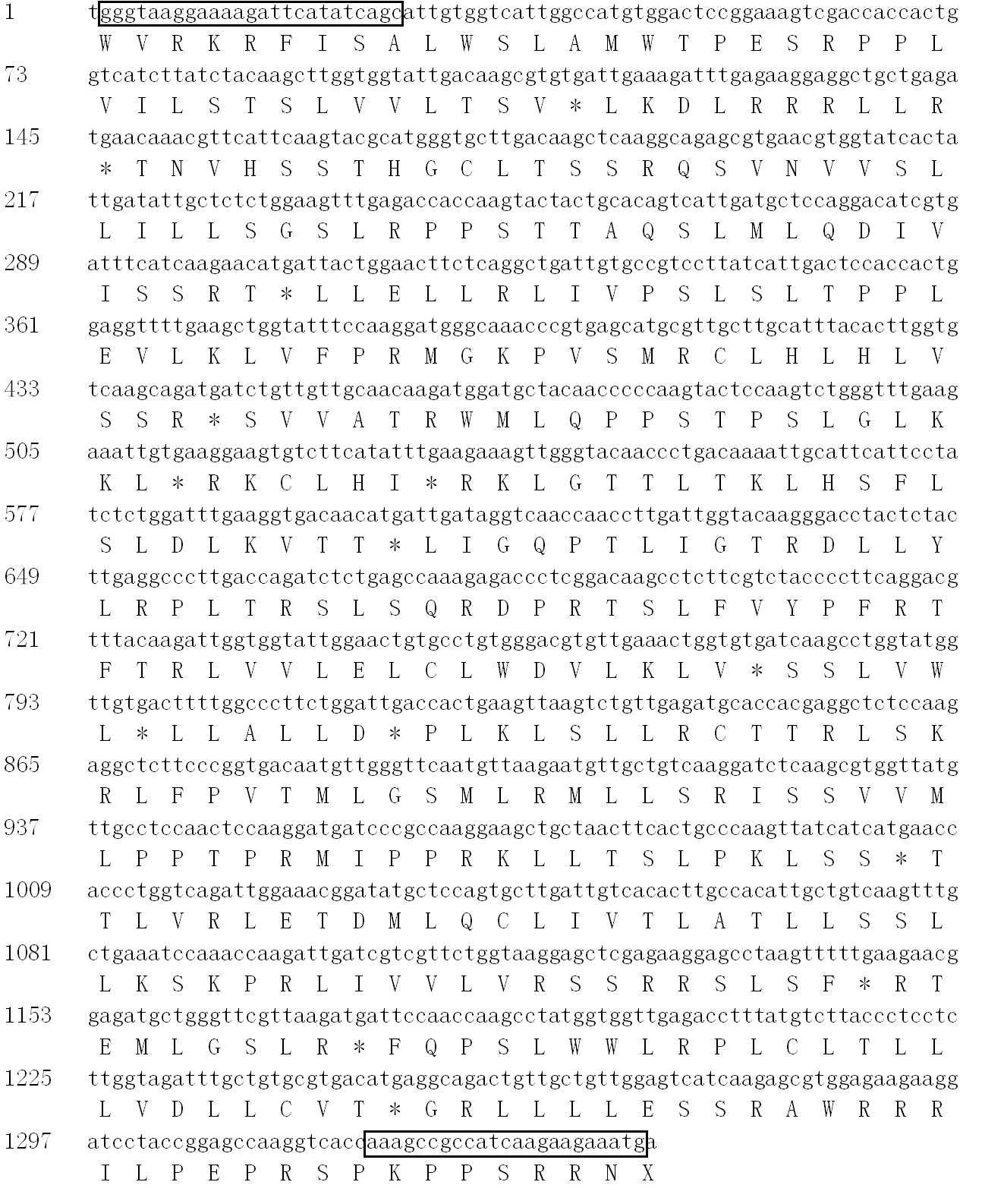


**Fig. K.**

Nucleotide acid and deduced amino acid sequences of *TUB* from carrot.


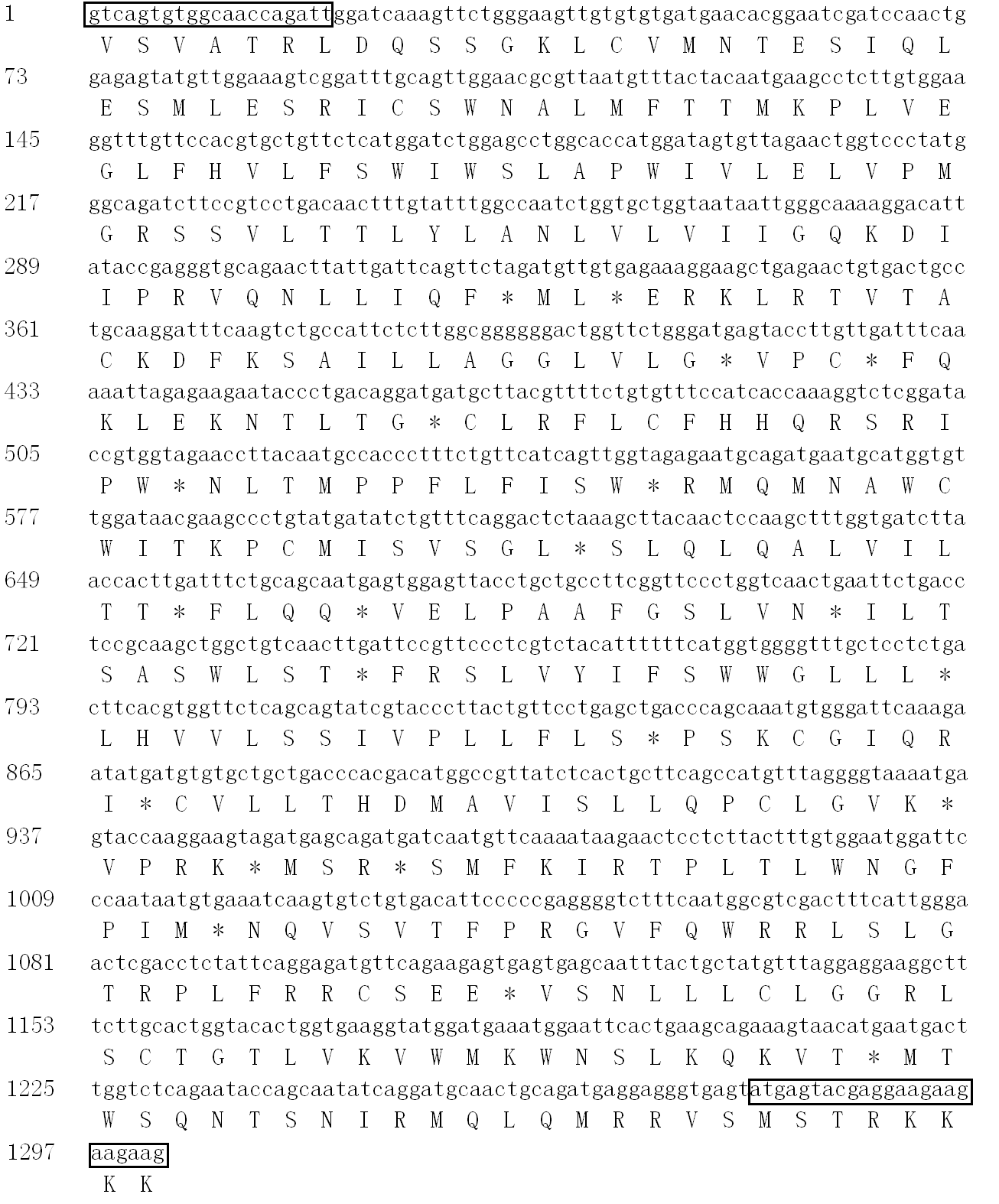


**Fig. L.**

Standard curves of each candidate genes.

Standard curves for *GAPDH*, *ACTIN*, *eIF-4α*, *PP2A*, *SAND*, *TIP41*, *UBQ*, *EF-1α*, and *TUB*. The linear correlation (R^2^) and PCR efficiencies (% E = (10^[−1/slope]^ - 1) × 100%) were calculated from the standard curve.


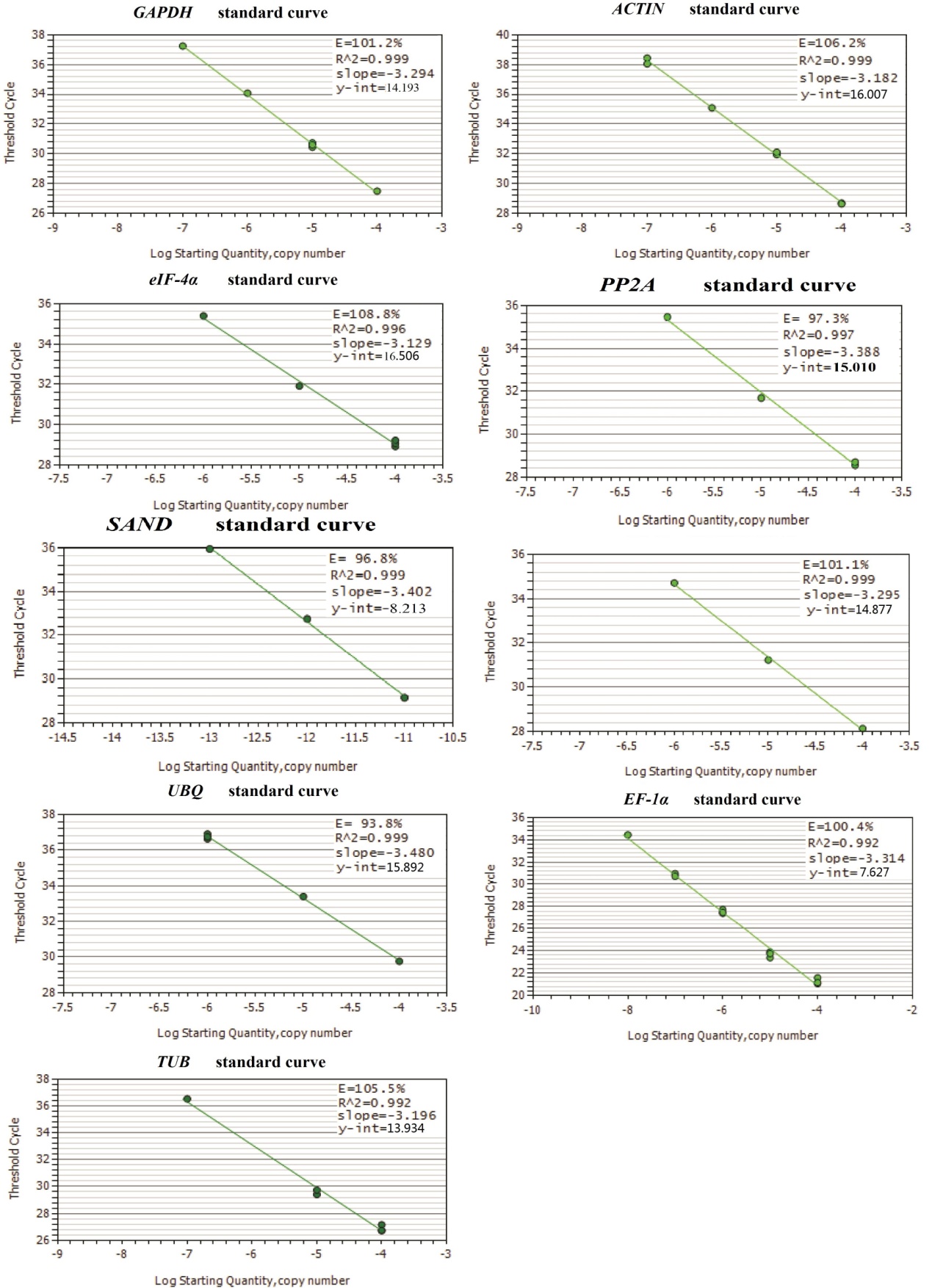


**Table A** Primer sequences for clone of nine reference genes from carrot.

| **Gene symbol** | **Primer sequence (5ʹ–3ʹ) forward/reverse** | **Amplicon length (bp)** |
| --- | --- | --- |
| *ACTIN* | ATGGCCGATGCCGAGGATAT/  GAAGCACTTCCTGTGCACAATA | 1,131 |
| *eIF-4α* | GCTGGAGCTGCACCTGAAGG/  CATCCTATCATCATCCTTGGTCAC | 1,166 |
| *GAPDH* | ATGCAGTCTTTAGTTGCAGC/  TTCAAAAACTTTGCACTCCTCTTCA | 1,374 |
| *PP2A* | TTGGATATGTGTTGCGTGTTG/  ACCCTAACCCTAATGGATGAATAGA | 935 |
| *SAND* | CATGCCTACTCTTGTCTCC/  TGTGCGGACCAAGTTCTCTAT | 709 |
| *TIP41* | GGAGGACTGTGAGGAACGAAT/  TTGGCTGATGCTGGTTGGAT | 355 |
| *UBQ* | ATGCAGATCTTTGTGAAAACTCTG/  TCAGAAATTGCCACCACGGA | 1,605 |
| *EF-1α* | GGGTAAGGAAAAGATTCATATCAGC/  CATTTCTTCTTGATGGCGGCTTT | 1,343 |
| *TUB* | GTCAGTGTGGCAACCAGATT/  CTTCTTCTTCTTCCTCGTACTCAT | 1,302 |

**Table B.** Raw Cq values in carrot.

Plants were subjected to the following stress treatments: heat, cold, drought, salt, SA, GA, MeJA, and ABA.

|  | *GAPDH* | *ACTIN* | *eIF-4α* | *PP2A* | *SAND* | *TIP41* | *UBQ* | *EF-1α* | *TUB* |
| --- | --- | --- | --- | --- | --- | --- | --- | --- | --- |
| Heat-1 | 25.72 | 27.34 | 31.01 | 32.23 | 33.07 | 34.34 | 27.73 | 22.49 | 31.90 |
| Heat-2 | 25.74 | 26.66 | 30.51 | 31.94 | 33.24 | 34.21 | 27.91 | 22.91 | 32.14 |
| Heat-3 | 24.84 | 26.31 | 30.93 | 32.26 | 33.91 | 35.31 | 27.92 | 21.94 | 31.28 |
| Heat-4 | 25.86 | 27.55 | 33.00 | 33.74 | 34.73 | 36.68 | 30.75 | 23.94 | 32.95 |
| Heat-5 | 26.29 | 26.85 | 31.35 | 34.99 | 35.03 | 36.10 | 29.44 | 23.38 | 32.96 |
| Heat-6 | 26.54 | 26.60 | 31.76 | 33.64 | 34.49 | 37.14 | 28.62 | 23.62 | 32.87 |
| Heat-7 | 24.61 | 24.89 | 29.14 | 31.68 | 31.70 | 33.21 | 27.74 | 22.46 | 29.88 |
| Heat-8 | 24.80 | 25.15 | 29.33 | 31.66 | 32.15 | 33.25 | 27.27 | 22.11 | 30.73 |
| Heat-9 | 23.71 | 25.34 | 28.67 | 30.88 | 31.16 | 33.69 | 27.04 | 22.09 | 30.85 |
| Cold-1 | 25.73 | 25.05 | 26.74 | 29.95 | 31.31 | 30.80 | 27.05 | 23.07 | 30.00 |
| Cold-2 | 25.70 | 25.04 | 27.03 | 30.43 | 31.38 | 30.75 | 26.85 | 22.92 | 30.45 |
| Cold-3 | 25.82 | 25.64 | 26.95 | 30.46 | 31.12 | 30.63 | 27.01 | 23.52 | 28.73 |
| Cold-4 | 26.93 | 24.91 | 27.62 | 30.66 | 30.89 | 33.12 | 26.87 | 22.47 | 29.70 |
| Cold-5 | 26.47 | 24.85 | 27.54 | 30.50 | 30.18 | 33.84 | 26.47 | 21.91 | 29.32 |
| Cold-6 | 25.97 | 24.81 | 27.36 | 30.59 | 31.18 | 32.32 | 26.40 | 22.77 | 29.75 |
| Cold-7 | 27.92 | 27.75 | 30.83 | 32.13 | 33.59 | 34.65 | 29.76 | 25.14 | 32.59 |
| Cold-8 | 29.03 | 28.04 | 31.42 | 31.57 | 30.67 | 32.29 | 29.77 | 25.27 | 31.83 |
| Cold-9 | 28.61 | 27.93 | 30.51 | 33.57 | 31.09 | 34.72 | 29.44 | 25.12 | 31.04 |
| Drought-1 | 25.08 | 25.28 | 26.61 | 31.15 | 30.50 | 31.65 | 27.17 | 22.81 | 29.51 |
| Drought-2 | 25.30 | 25.82 | 25.61 | 30.44 | 31.27 | 31.13 | 27.19 | 23.03 | 29.12 |
| Drought-3 | 25.19 | 25.00 | 27.52 | 30.36 | 30.33 | 31.31 | 27.21 | 22.82 | 28.65 |
| Drought-4 | 24.52 | 24.64 | 26.59 | 30.50 | 31.65 | 31.69 | 26.76 | 22.92 | 29.43 |
| Drought-5 | 24.65 | 24.93 | 26.69 | 30.92 | 31.17 | 30.56 | 26.88 | 23.18 | 29.03 |
| Drought-6 | 25.12 | 25.33 | 27.03 | 30.12 | 31.62 | 31.16 | 26.99 | 23.51 | 29.37 |
| Drought-7 | 21.26 | 21.61 | 22.61 | 25.67 | 27.00 | 24.97 | 22.88 | 18.62 | 26.34 |
| Drought-8 | 21.29 | 21.23 | 22.01 | 25.27 | 26.66 | 24.79 | 22.69 | 18.66 | 26.02 |
| Drought-9 | 20.40 | 20.99 | 22.09 | 25.38 | 26.88 | 24.42 | 21.91 | 18.76 | 26.12 |
| Salt-1 | 27.43 | 27.66 | 29.58 | 31.91 | 31.39 | 34.43 | 28.72 | 24.11 | 30.36 |
| Salt-2 | 27.33 | 27.24 | 30.33 | 31.45 | 31.39 | 32.37 | 28.45 | 24.10 | 29.94 |
| Salt-3 | 27.21 | 27.75 | 30.21 | 32.78 | 35.63 | 33.51 | 28.70 | 24.09 | 29.30 |
| Salt-4 | 25.95 | 25.38 | 32.17 | 32.65 | 31.02 | 31.93 | 28.73 | 22.61 | 30.33 |
| Salt-5 | 26.49 | 25.65 | 30.69 | 30.96 | 30.63 | 32.79 | 28.25 | 22.35 | 32.78 |
| Salt-6 | 26.29 | 24.82 | 30.92 | 30.53 | 33.90 | 31.99 | 28.26 | 22.14 | 31.24 |
| Salt-7 | 27.15 | 27.49 | 30.31 | 32.66 | 30.11 | 33.68 | 28.76 | 24.09 | 30.66 |
| Salt-8 | 27.45 | 27.34 | 29.53 | 31.44 | 31.71 | 31.67 | 29.15 | 23.81 | 30.42 |
| Salt-9 | 28.14 | 28.55 | 30.89 | 30.88 | 33.59 | 33.82 | 29.71 | 24.94 | 31.36 |
| SA-1 | 28.19 | 28.83 | 31.44 | 34.34 | 34.04 | 33.73 | 30.58 | 26.12 | 32.70 |
| SA-2 | 28.96 | 28.95 | 31.29 | 34.13 | 33.60 | 32.43 | 29.97 | 25.98 | 32.35 |
| SA-3 | 27.89 | 28.86 | 30.64 | 34.45 | 34.45 | 33.32 | 29.62 | 25.24 | 32.65 |
| SA-4 | 27.87 | 28.68 | 29.65 | 33.59 | 33.11 | 33.56 | 28.37 | 25.63 | 32.08 |
| SA-5 | 27.84 | 28.71 | 29.80 | 33.72 | 35.35 | 32.78 | 28.14 | 25.79 | 31.21 |
| SA-6 | 27.92 | 28.78 | 29.63 | 30.66 | 33.36 | 33.91 | 29.27 | 25.41 | 32.82 |
| SA-7 | 29.30 | 28.98 | 30.66 | 31.33 | 30.27 | 32.25 | 30.11 | 25.54 | 32.62 |
| SA-8 | 27.72 | 29.44 | 29.84 | 32.25 | 30.80 | 33.64 | 30.24 | 25.38 | 33.55 |
| SA-9 | 28.37 | 28.64 | 29.17 | 32.46 | 32.47 | 31.82 | 31.17 | 25.22 | 33.90 |
| GA-1 | 25.00 | 26.90 | 28.37 | 31.65 | 31.99 | 33.31 | 29.73 | 23.21 | 32.23 |
| GA-2 | 25.60 | 27.19 | 28.31 | 31.70 | 33.67 | 31.91 | 30.51 | 23.62 | 31.13 |
| GA-3 | 26.11 | 27.92 | 29.64 | 31.90 | 33.12 | 32.64 | 30.50 | 23.92 | 31.07 |
| GA-4 | 25.63 | 26.74 | 28.56 | 31.87 | 33.45 | 32.38 | 30.19 | 23.54 | 30.83 |
| GA-5 | 25.60 | 25.98 | 28.64 | 30.18 | 32.95 | 31.22 | 29.84 | 23.95 | 30.00 |
| GA-6 | 25.79 | 25.86 | 28.41 | 29.82 | 32.29 | 31.51 | 29.65 | 23.73 | 30.78 |
| GA-7 | 25.63 | 26.93 | 28.49 | 29.72 | 32.72 | 32.14 | 29.67 | 23.61 | 29.91 |
| GA-8 | 25.71 | 26.98 | 28.74 | 29.35 | 31.42 | 33.61 | 28.90 | 22.43 | 29.89 |
| GA-9 | 25.83 | 26.02 | 28.92 | 30.64 | 33.54 | 32.41 | 30.05 | 22.80 | 30.55 |
| ABA-1 | 29.47 | 31.76 | 33.02 | 30.27 | 37.14 | 33.61 | 32.79 | 31.11 | 33.52 |
| ABA-2 | 30.42 | 32.64 | 35.13 | 30.01 | 33.53 | 38.01 | 32.65 | 30.81 | 32.22 |
| ABA-3 | 30.16 | 33.02 | 31.39 | 30.83 | 32.62 | 36.68 | 34.90 | 29.67 | 34.22 |
| ABA-4 | 29.86 | 32.76 | 33.20 | 32.12 | 33.29 | 35.05 | 32.10 | 29.79 | 33.17 |
| ABA-5 | 30.74 | 32.77 | 32.41 | 32.11 | 35.17 | 36.59 | 33.54 | 30.59 | 34.40 |
| ABA-6 | 29.31 | 31.36 | 31.85 | 32.03 | 32.84 | 36.62 | 33.88 | 29.72 | 33.73 |
| ABA-7 | 30.19 | 32.53 | 33.35 | 33.69 | 32.64 | 35.70 | 33.55 | 30.60 | 32.88 |
| ABA-8 | 28.84 | 31.45 | 34.23 | 33.06 | 32.69 | 32.10 | 33.38 | 29.98 | 33.37 |
| ABA-9 | 29.96 | 30.58 | 34.39 | 32.68 | 33.66 | 37.27 | 32.46 | 28.58 | 33.19 |
| MeJA-1 | 26.90 | 26.10 | 29.19 | 31.79 | 31.47 | 31.98 | 26.76 | 24.00 | 30.03 |
| MeJA-2 | 26.90 | 26.48 | 29.85 | 32.27 | 31.62 | 32.63 | 26.73 | 23.82 | 31.08 |
| MeJA-3 | 26.82 | 25.31 | 29.66 | 31.52 | 31.81 | 31.39 | 26.83 | 24.14 | 30.53 |
| MeJA-4 | 29.41 | 29.66 | 31.50 | 32.60 | 33.25 | 32.63 | 29.07 | 26.25 | 32.11 |
| MeJA-5 | 28.54 | 29.01 | 30.47 | 31.01 | 32.15 | 34.83 | 29.10 | 26.33 | 32.02 |
| MeJA-6 | 29.89 | 29.16 | 31.69 | 32.51 | 36.53 | 33.37 | 29.18 | 26.61 | 33.51 |
| MeJA-7 | 26.84 | 26.26 | 28.80 | 31.63 | 31.84 | 32.23 | 28.47 | 25.23 | 33.32 |
| MeJA-8 | 27.67 | 26.81 | 29.21 | 31.02 | 31.74 | 33.90 | 28.67 | 25.62 | 31.38 |
| MeJA-9 | 27.30 | 27.11 | 29.12 | 32.06 | 31.48 | 34.95 | 28.78 | 25.68 | 30.44 |

**Table C** Gene expression stability in carrot under individual stress conditions, as ranked by the three software programs geNorm, NormFinder, and BestKeeper.

Plants were subjected to the following stress treatments: heat, cold, salt, drought, SA, GA, MeJA, and ABA.

| **Treatments** | **Rank** | **geNorm** |  |  | **NormFinder** |  |  | **BestKeeper** |  |  |
| --- | --- | --- | --- | --- | --- | --- | --- | --- | --- | --- |
|  |  | **Gene** | **Stability** |  | **Gene** | **Stability** |  | **Gene** | **SD** | **CV** |
| Heat | 1 | *eIF-4α* | 0.55 |  | *SAND* | 0.004 |  | *EF-1α* | 0.62 | 2.70 |
|  | 2 | *SAND* | 0.55 |  | *UBQ* | 0.005 |  | *GAPDH* | 0.76 | 3.00 |
|  | 3 | *TUB* | 0.64 |  | *PP2A* | 0.007 |  | *ACTIN* | 0.78 | 2.97 |
|  | 4 | *ACTIN* | 0.66 |  | *eIF-4α* | 0.010 |  | *UBQ* | 0.89 | 3.15 |
|  | 5 | *GAPDH* | 0.68 |  | *ACTIN* | 0.012 |  | *TUB* | 0.93 | 2.93 |
|  | 6 | *EF-1α* | 0.71 |  | *TUB* | 0.012 |  | *PP2A* | 1.04 | 3.21 |
|  | 7 | *UBQ* | 0.73 |  | *TIP41* | 0.013 |  | *eIF-4α* | 1.09 | 3.55 |
|  | 8 | *PP2A* | 0.74 |  | *GAPDH* | 0.018 |  | *SAND* | 1.12 | 3.37 |
|  | 9 | *TIP41* | 0.77 |  | *EF-1α* | 0.021 |  | *TIP41* | 1.27 | 3.63 |
| Cold | 1 | *ACTIN* | 0.23 |  | *UBQ* | 0.005 |  | *SAND* | 0.55 | 1.76 |
|  | 2 | *UBQ* | 0.23 |  | *eIF-4α* | 0.007 |  | *PP2A* | 0.88 | 2.84 |
|  | 3 | *EF-1α* | 0.34 |  | *PP2A* | 0.008 |  | *TUB* | 0.98 | 3.21 |
|  | 4 | *GAPDH* | 0.51 |  | *GAPDH* | 0.010 |  | *EF-1α* | 1.07 | 4.52 |
|  | 5 | *eIF-4α* | 0.61 |  | *ACTIN* | 0.015 |  | *GAPDH* | 1.08 | 4.01 |
|  | 6 | *PP2A* | 0.69 |  | *TIP41* | 0.015 |  | *ACTIN* | 1.27 | 4.88 |
|  | 7 | *TUB* | 0.74 |  | *SAND* | 0.020 |  | *UBQ* | 1.28 | 4.62 |
|  | 8 | *SAND* | 0.89 |  | *EF-1α* | 0.020 |  | *TIP41* | 1.35 | 4.13 |
|  | 9 | *TIP41* | 1.02 |  | *TUB* | 0.036 |  | *eIF-4α* | 1.65 | 5.81 |
|  | 1 | *GAPDH* | 0.25 |  | *ACTIN* | 0.018 |  | *TUB* | 1.34 | 4.77 |
|  | 2 | *ACTIN* | 0.25 |  | *TIP41* | 0.024 |  | *ACTIN* | 1.73 | 7.25 |
|  | 3 | *UBQ* | 0.33 |  | *TUB* | 0.041 |  | *GAPDH* | 1.78 | 7.51 |
|  | 4 | *EF-1α* | 0.39 |  | *eIF-4α* | 0.056 |  | *SAND* | 1.89 | 6.35 |
| Drought | 5 | *SAND* | 0.46 |  | *PP2A* | 0.057 |  | *EF-1α* | 1.94 | 8.98 |
|  | 6 | *eIF-4α* | 0.53 |  | *SAND* | 0.069 |  | *eIF-4α* | 1.97 | 7.83 |
|  | 7 | *PP2A* | 0.58 |  | *EF-1α* | 0.112 |  | *UBQ* | 2.02 | 7.90 |
|  | 8 | *TUB* | 0.64 |  | *UBQ* | 0.115 |  | *PP2A* | 2.29 | 7.92 |
|  | 9 | *TIP41* | 0.78 |  | *GAPDH* | 0.128 |  | *TIP41* | 2.90 | 9.97 |
| Salt | 1 | *ACTIN* | 0.36 |  | *TIP41* | 0.001 |  | *UBQ* | 0.31 | 1.07 |
|  | 2 | *EF-1α* | 0.36 |  | *UBQ* | 0.001 |  | *GAPDH* | 0.54 | 1.98 |
|  | 3 | *GAPDH* | 0.48 |  | *GAPDH* | 0.002 |  | *eIF-4α* | 0.58 | 1.91 |
|  | 4 | *UBQ* | 0.61 |  | *eIF-4α* | 0.004 |  | *PP2A* | 0.72 | 2.26 |
|  | 5 | *TIP41* | 0.73 |  | *PP2A* | 0.008 |  | *TUB* | 0.72 | 2.35 |
|  | 6 | *PP2A* | 0.87 |  | *ACTIN* | 0.019 |  | *EF-1α* | 0.81 | 3.43 |
|  | 7 | *eIF-4α* | 1.01 |  | *EF-1α* | 0.025 |  | *TIP41* | 0.84 | 2.56 |
|  | 8 | *TUB* | 1.13 |  | *SAND* | 0.031 |  | *ACTIN* | 1.06 | 3.95 |
|  | 9 | *SAND* | 1.32 |  | *TUB* | 0.032 |  | *SAND* | 1.48 | 4.61 |
| SA | 1 | *ACTIN* | 0.42 |  | *TIP41* | 0.001 |  | *ACTIN* | 0.16 | 0.57 |
|  | 2 | *EF-1α* | 0.42 |  | *EF-1α* | 0.001 |  | *EF-1α* | 0.26 | 1.01 |
|  | 3 | *GAPDH* | 0.53 |  | *eIF-4α* | 0.001 |  | *GAPDH* | 0.43 | 1.53 |
|  | 4 | *eIF-4α* | 0.62 |  | *ACTIN* | 0.001 |  | *TUB* | 0.52 | 1.61 |
|  | 5 | *TUB* | 0.76 |  | *GAPDH* | 0.002 |  | *TIP41* | 0.65 | 1.96 |
|  | 6 | *UBQ* | 0.82 |  | *UBQ* | 0.005 |  | *eIF-4α* | 0.69 | 2.27 |
|  | 7 | *TIP41* | 0.88 |  | *PP2A* | 0.006 |  | *UBQ* | 0.77 | 2.60 |
|  | 8 | *PP2A* | 1.04 |  | *TUB* | 0.008 |  | *PP2A* | 1.17 | 3.55 |
|  | 9 | *SAND* | 1.21 |  | *SAND* | 0.028 |  | *SAND* | 1.25 | 3.77 |
| GA | 1 | *GAPDH* | 0.29 |  | *UBQ* | 0.003 |  | *GAPDH* | 0.18 | 0.72 |
|  | 2 | *eIF-4α* | 0.29 |  | *eIF-4α* | 0.006 |  | *eIF-4α* | 0.28 | 0.99 |
|  | 3 | *UBQ* | 0.45 |  | *TIP41* | 0.006 |  | *UBQ* | 0.37 | 1.25 |
|  | 4 | *EF-1α* | 0.50 |  | *SAND* | 0.007 |  | *EF-1α* | 0.41 | 1.75 |
|  | 5 | *SAND* | 0.57 |  | *GAPDH* | 0.009 |  | *ACTIN* | 0.51 | 1.92 |
|  | 6 | *ACTIN* | 0.63 |  | *ACTIN* | 0.009 |  | *TUB* | 0.55 | 1.80 |
|  | 7 | *TUB* | 0.70 |  | *EF-1α* | 0.014 |  | *TIP41* | 0.58 | 1.79 |
|  | 8 | *PP2A* | 0.75 |  | *PP2A* | 0.014 |  | *SAND* | 0.61 | 1.87 |
|  | 9 | *TIP41* | 0.81 |  | *TUB* | 0.017 |  | *PP2A* | 0.91 | 2.95 |
| ABA | 1 | *GAPDH* | 0.67 |  | *ACTIN* | 0.000 |  | *GAPDH* | 0.46 | 1.53 |
|  | 2 | *ACTIN* | 0.67 |  | *EF-1α* | 0.000 |  | *TUB* | 0.49 | 1.48 |
|  | 3 | *TUB* | 0.86 |  | *UBQ* | 0.000 |  | *EF-1α* | 0.61 | 2.02 |
|  | 4 | *UBQ* | 0.88 |  | *eIF-4α* | 0.000 |  | *UBQ* | 0.67 | 2.01 |
|  | 5 | *EF-1α* | 0.92 |  | *GAPDH* | 0.000 |  | *ACTIN* | 0.72 | 2.24 |
|  | 6 | *PP2A* | 1.14 |  | *TIP41* | 0.002 |  | *eIF-4α* | 0.94 | 2.82 |
|  | 7 | *eIF-4α* | 1.28 |  | *TUB* | 0.002 |  | *PP2A* | 1.00 | 3.14 |
|  | 8 | *SAND* | 1.41 |  | *SAND* | 0.006 |  | *SAND* | 1.08 | 3.19 |
|  | 9 | *TIP41* | 1.57 |  | *PP2A* | 0.013 |  | *TIP41* | 1.44 | 4.03 |
| MeJA | 1 | *UBQ* | 0.21 |  | *GAPDH* | 0.003 |  | *PP2A* | 0.48 | 1.50 |
|  | 2 | *EF-1α* | 0.21 |  | *EF-1α* | 0.004 |  | *eIF-4α* | 0.85 | 2.84 |
|  | 3 | *GAPDH* | 0.57 |  | *TIP41* | 0.006 |  | *EF-1α* | 0.89 | 3.52 |
|  | 4 | *ACTIN* | 0.69 |  | *eIF-4α* | 0.006 |  | *UBQ* | 0.94 | 3.32 |
|  | 5 | *eIF-4α* | 0.76 |  | *SAND* | 0.007 |  | *GAPDH* | 0.98 | 3.53 |
|  | 6 | *TUB* | 0.88 |  | *UBQ* | 0.008 |  | *TUB* | 1.01 | 3.20 |
|  | 7 | *PP2A* | 0.97 |  | *PP2A* | 0.009 |  | *TIP41* | 1.03 | 3.12 |
|  | 8 | *SAND* | 1.03 |  | *ACTIN* | 0.010 |  | *SAND* | 1.09 | 3.37 |
|  | 9 | *TIP41* | 1.12 |  | *TUB* | 0.016 |  | *ACTIN* | 1.30 | 4.77 |
